# Conservation of IAMT Preference for Indole Acetic Acid Methylation Across 250 Million Years of Seed Plant Divergence, With Only One Recent Evolutionary Switch in *Ocimum*

**DOI:** 10.1093/gbe/evag167

**Published:** 2026-07-02

**Authors:** Bahar Saadaie Jahromi, Liberty M Kostrzewa, Turki Alhinaai, Tariq Al-Zadjali, Afrida Amin, Allyson Barth, McKenna E Bayne, Gregory R Begeman, William H Bell, Melanie G Bucko, Emily M Catania, Angel Currie, Glaucia Fernanda de Lima Pereira, Citlali Figueroa, Allexia B Galentine, Gedi Gambrell, Fernando D Garcia, Avery E Gauthier, Joseph R Graber, Brenna J Hagenbarth, Amethyst Hall, Madeline Heemstra, Ruiqi Huang, Johnathon Hunter, Ami Jaswa, Nathania Karyadi, Caleb J Learman, Kar Men Lee, Clayton T Lewis, Elizabeth McKenzie, Marie F McKinney, Samantha McLean, Haley M Mueller, Merub Nadeem, Sneha Nath, Vyctoria S Nusbaum, Bryan O'Connor, Nivetha Pasupathy, Enish Pathak, Tashfia Tahsin Raisa, Karina Ramirez, Bridget Salazar, Christian E Sander, Erin Schneider, Kevin Sedlacek, Gwenyth Sirrine, Jake B Spitsbergen, Leire Garcia Torres, My Hien Truong, Brisa Hernandez Velasco, Royal Woolfolk, Todd J Barkman

**Affiliations:** Department of Biological Sciences, Western Michigan University, Kalamazoo, MI 49008, USA; Department of Biological Sciences, Western Michigan University, Kalamazoo, MI 49008, USA; Department of Biological Sciences, Western Michigan University, Kalamazoo, MI 49008, USA; Department of Biological Sciences, Western Michigan University, Kalamazoo, MI 49008, USA; Department of Biological Sciences, Western Michigan University, Kalamazoo, MI 49008, USA; Department of Biological Sciences, Western Michigan University, Kalamazoo, MI 49008, USA; Department of Biological Sciences, Western Michigan University, Kalamazoo, MI 49008, USA; Department of Biological Sciences, Western Michigan University, Kalamazoo, MI 49008, USA; Department of Biological Sciences, Western Michigan University, Kalamazoo, MI 49008, USA; Department of Biological Sciences, Western Michigan University, Kalamazoo, MI 49008, USA; Department of Biological Sciences, Western Michigan University, Kalamazoo, MI 49008, USA; Department of Biological Sciences, Western Michigan University, Kalamazoo, MI 49008, USA; Department of Biological Sciences, Western Michigan University, Kalamazoo, MI 49008, USA; Department of Biological Sciences, Western Michigan University, Kalamazoo, MI 49008, USA; Department of Biological Sciences, Western Michigan University, Kalamazoo, MI 49008, USA; Department of Biological Sciences, Western Michigan University, Kalamazoo, MI 49008, USA; Department of Biological Sciences, Western Michigan University, Kalamazoo, MI 49008, USA; Department of Biological Sciences, Western Michigan University, Kalamazoo, MI 49008, USA; Department of Biological Sciences, Western Michigan University, Kalamazoo, MI 49008, USA; Department of Biological Sciences, Western Michigan University, Kalamazoo, MI 49008, USA; Department of Biological Sciences, Western Michigan University, Kalamazoo, MI 49008, USA; Department of Biological Sciences, Western Michigan University, Kalamazoo, MI 49008, USA; Department of Biological Sciences, Western Michigan University, Kalamazoo, MI 49008, USA; Department of Biological Sciences, Western Michigan University, Kalamazoo, MI 49008, USA; Department of Biological Sciences, Western Michigan University, Kalamazoo, MI 49008, USA; Department of Biological Sciences, Western Michigan University, Kalamazoo, MI 49008, USA; Department of Biological Sciences, Western Michigan University, Kalamazoo, MI 49008, USA; Department of Biological Sciences, Western Michigan University, Kalamazoo, MI 49008, USA; Department of Biological Sciences, Western Michigan University, Kalamazoo, MI 49008, USA; Department of Biological Sciences, Western Michigan University, Kalamazoo, MI 49008, USA; Department of Biological Sciences, Western Michigan University, Kalamazoo, MI 49008, USA; Department of Biological Sciences, Western Michigan University, Kalamazoo, MI 49008, USA; Department of Biological Sciences, Western Michigan University, Kalamazoo, MI 49008, USA; Department of Biological Sciences, Western Michigan University, Kalamazoo, MI 49008, USA; Department of Biological Sciences, Western Michigan University, Kalamazoo, MI 49008, USA; Department of Biological Sciences, Western Michigan University, Kalamazoo, MI 49008, USA; Department of Biological Sciences, Western Michigan University, Kalamazoo, MI 49008, USA; Department of Biological Sciences, Western Michigan University, Kalamazoo, MI 49008, USA; Department of Biological Sciences, Western Michigan University, Kalamazoo, MI 49008, USA; Department of Biological Sciences, Western Michigan University, Kalamazoo, MI 49008, USA; Department of Biological Sciences, Western Michigan University, Kalamazoo, MI 49008, USA; Department of Biological Sciences, Western Michigan University, Kalamazoo, MI 49008, USA; Department of Biological Sciences, Western Michigan University, Kalamazoo, MI 49008, USA; Department of Biological Sciences, Western Michigan University, Kalamazoo, MI 49008, USA; Department of Biological Sciences, Western Michigan University, Kalamazoo, MI 49008, USA; Department of Biological Sciences, Western Michigan University, Kalamazoo, MI 49008, USA; Department of Biological Sciences, Western Michigan University, Kalamazoo, MI 49008, USA; Department of Biological Sciences, Western Michigan University, Kalamazoo, MI 49008, USA; Department of Biological Sciences, Western Michigan University, Kalamazoo, MI 49008, USA; Department of Biological Sciences, Western Michigan University, Kalamazoo, MI 49008, USA; Department of Biological Sciences, Western Michigan University, Kalamazoo, MI 49008, USA; Department of Biological Sciences, Western Michigan University, Kalamazoo, MI 49008, USA

**Keywords:** molecular evolution, enzyme evolution, plant specialized metabolism

## Abstract

Plant indole-3-acetic acid methyltransferase (IAMT) is an ancient SABATH enzyme that modulates auxin levels via S-adenosyl-L-methionine (SAM)–dependent methylation. While most IAMTs studied previously prefer to methylate indole-3-acetic acid (IAA), one orthologous enzyme prefers to methylate cinnamic acid (CA) and shows no activity toward IAA. To understand whether other closely related enzymes also show substrate preference switches, we combined molecular phylogenetic analyses with in vitro enzyme assays. A maximum likelihood tree of 704 IAMT-like enzymes shows pervasive retention of IAMT orthologs across nearly all angiosperms and gymnosperms. Our newly reported enzymatic assays of 59 orthologs spanning 52 species and 26 orders revealed strongly conserved methylation preference for IAA across all lineages. However, in the genus *Ocimum* (Lamiaceae), species appear to possess one copy of IAMT that encodes an IAA-preferring enzyme and a second copy that encodes an enzyme that prefers to methylate CA. Altogether, we experimentally investigated four lineages, Lamiales, Solanales, Fabales, and Rosales, in which IAMT-type enzymes were duplicated but no others showed evidence for substrate preference switches. Alignments and computational modeling of Lamiaceae sequences highlighted M35, L325, and L363 as amino acid substitutions that may account for CA preference evolution in *Ocimum* enzymes. Yet, experimental mutation of those sites in either modern-day or ancestral sequences did not result in substrate preference changes in enzyme assays. Therefore, further studies are required to phylogenetically pinpoint the timing and structural basis for the shift toward CA methylation preference and to deepen our understanding of molecular evolution of the SABATH enzyme family.

Significance Enzymes drive essential functions throughout the tree of life but are rarely studied broadly outside of model species. Here, we investigate substrate preference evolution throughout the flowering plant clade using enzyme assays from 59 IAMT orthologs and show that preference for indole-3-acetic acid is maintained across all species. Though many duplicates were functionally investigated, only in one lineage was evolution of a substrate preference switch to cinnamic acid found. Mutation of three active site amino acid residues did not result in a substrate preference switch. Therefore, additional sites, that may or may not be epistatic with the three active site residues, must be responsible for this evolutionary change.

## Introduction

Enzymes are nature's catalysts, driving the countless biochemical reactions that sustain life. Most enzymes belong to large gene families that have expanded through gene duplication, an evolutionary mechanism that allows organisms to acquire new biochemical functions. It is posited that, after duplication, the daughter genes may retain ancestral roles, or one or both duplicates can accumulate mutations that potentially lead to novel functions (Ohno 1970; Force et al. 1999; Bergthorsson et al. 2007; Des Marais and Rausher 2008). This process is thought to have generated myriad enzymes that produce the vast diversity of plant specialized metabolites, which play important roles in regulating growth and development as well as environmental interactions, including defense against herbivores and pathogens, attraction of pollinators and seed dispersers, and responses to abiotic stress (Pichersky and Gang 2000; Seo et al. 2001; Howe and Jander 2008; Vlot et al. 2009).

**Fig. 1.**
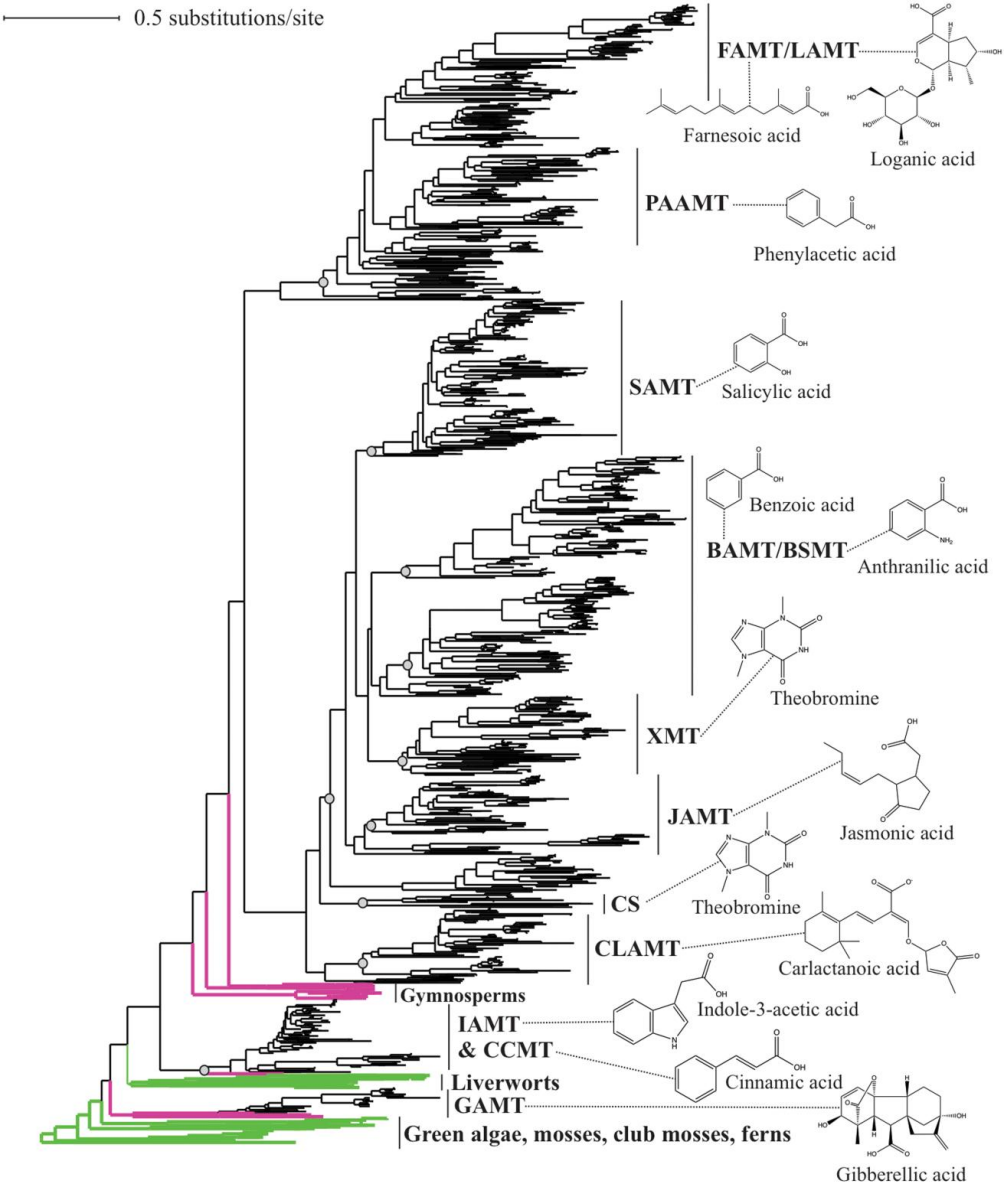
Phylogenetic analysis of the SABATH family reveals relationships among enzymes that methylate a variety of plant hormones and secondary metabolites. Bootstrap support >80 is shown by circles for selected nodes that separate the IAA-methylating clade of enzymes from others. Branches corresponding to proteins from green algae, non-seed plants and gymnosperms are marked. All others are from angiosperms. Selected preferred substrate structures for members of each clade are depicted, highlighting the range of biochemicals modified by SABATH enzymes. SAMT, salicylic acid MT; BAMT, benzoic acid MT; BSMT, benzoic/salicylic acid MT; JMT, jasmonic acid MT; XMT, xanthine alkaloid MT; CS, caffeine synthase; IAMT, indole acetic acid MT; GAMT, gibberellic acid MT; FAMT, farnesoic acid MT; CCMT, cinnamic acid MT; LAMT, loganic acid MT; PAAMT, phenylacetic acid MT; CLAMT, carlactonoic acid MT. Tree modified from Dubs et al. (2022).

Specialized metabolites are often modified by enzymatic processes such as methylation (Noel et al. 2003). Methylation is frequently achieved by enzymatic transfer of a methyl group from S-adenosyl-L-methionine (SAM) to a methyl-acceptor substrate (Noel et al. 2003). The SABATH enzyme family is one group of plant methyltransferases that helps regulate growth, defense, and metabolism (Wang et al. 2024). SABATH methyltransferases include many members that have evolved distinct substrate preferences, such as salicylic acid methyltransferase (SAMT), benzoic acid methyltransferases (BAMT), jasmonic acid methyltransferases (JAMT), gibberellic acid methyltransferases (GAMT), and indole-3-acetic acid methyltransferases (IAMT), in addition to many others (Ross et al. 1999; Seo et al. 2001; Qin et al. 2005; Varbanova et al. 2007; Wang et al. 2024) (Fig. 1). The SABATH gene family has expanded significantly by gene duplication in seed plants because angiosperms appear to encode 24 to 41 copies while gymnosperms encode between 10 and 42 copies (D'Auria et al. 2003; Zhao et al. 2007, 2008; Wang et al. 2019, 2024; Zhuge et al. 2023). In contrast, non-vascular plants seem to possess fewer SABATH genes because only four copies are known in the moss *Physcomitrella patens* and only six in the liverwort, *Conocephalum salebrosum* (Zhao et al. 2012; Chaiprasongsuk et al. 2018). Fewer still are found in green algae with all known sequences composing a single lineage that is sister to the entire family of enzymes in land plants (Wang et al. 2024) (Fig. 1); however, the functions of these are unknown. Algal lineages have the biochemical potential to produce some substrates of modern-day SABATH enzymes (Kunz et al. 2024), so future experiments should investigate these in order to shed light on the extent to which SABATH enzyme diversification was linked to the colonization of land (Kunz et al. 2025). The history of the SABATH enzyme family demonstrates both recent evolution of lineage-specific enzymatic functions, such as that of caffeine biosynthesis, which is found in only a few disparate flowering plant lineages (Huang et al. 2016), as well as the ancient conservation of some functions, such as indole-3-acetic acid (IAA) methylation, which is found in gymnosperms and angiosperms (Zhao et al. 2008; Wang et al. 2024).

**Fig. 2.**
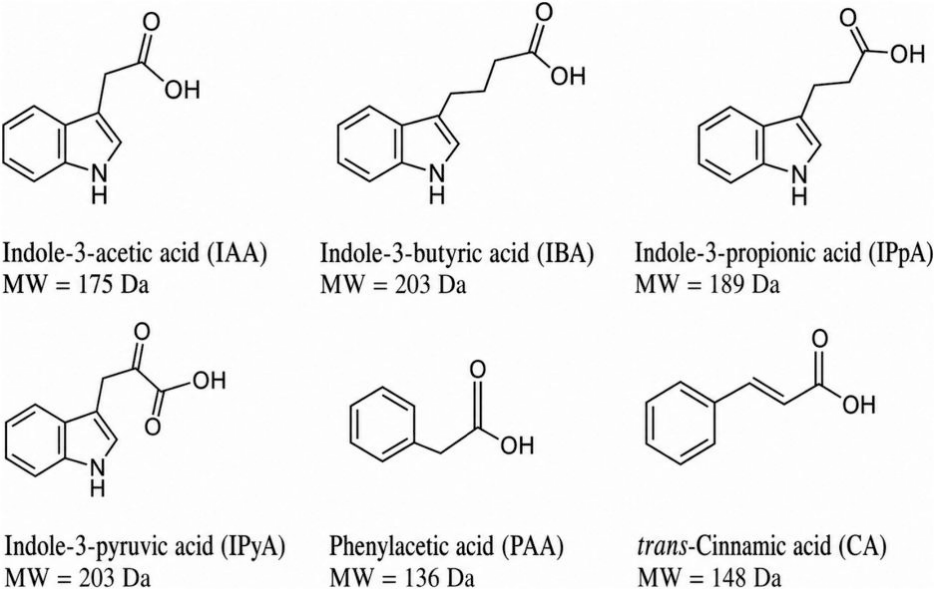
Chemical structures and molecular weights of land plant auxins and other plant metabolites. All six compounds were used as substrates in IAMT enzyme assays.

IAA is the primary auxin in land plants and a main regulator of processes such as cell elongation, apical dominance, tropisms, and organogenesis (Leyser 2002; Teale et al. 2006; Zhao 2010; Casanova-Sáez et al. 2021). Auxin homeostasis and regulation are achieved in several ways including biosynthesis, degradation, conjugation, and methylation (Qin et al. 2005; Ludwig-Müller 2011; Abbas et al. 2018). Methylation of IAA by the SABATH enzyme, IAMT, results from the transfer of a methyl group to the carboxylate moiety to form methyl-IAA (MeIAA). MeIAA is typically inactive or a storage form of auxin, though can be reactivated through hydrolysis (Zhao et al. 2008; Takubo et al. 2020). Although IAA is the most abundant and well-studied auxin in plants, it is not the only molecule exhibiting auxin-like activity. Phenylacetic acid (PAA), indole-3-propionic acid (IPpA), and indole-3-butyric acid (IBA) (Fig. 2) also exhibit auxin activity, playing roles in modulating growth and development to varying extents (Nordström et al. 1991; Ludwig-Müller and Cohen 2002; Ludwig-Müller 2011).

Studies of most IAMT enzymes have documented substrate preference for IAA methylation; yet, lower relative activity toward other auxin-like compounds has been observed. For instance, IAMT1 from *Arabidopsis thaliana* (AtIAMT1) catalyzes the methylation of both IAA and PAA in vitro (Takubo et al. 2020). In *Oryza sativa*, OsIAMT methylates IAA, IBA, and synthetic auxins (Zhao et al. 2008), pointing to broader substrate flexibility. Enzymes from *Populus trichocarpa* (PtIAMT) also strongly prefer IAA, though weak activity toward farnesoic acid (FA) has been observed (Zhao et al. 2007; Han et al. 2018). By contrast, *Illicium anisatum* IAMT exhibits high substrate specificity, with no detectable activity toward IBA or IPpA under assay conditions (Koeduka et al. 2024). While methylation preference for IAA by IAMT appears to be an anciently conserved trait, because it has been found in both angiosperms and gymnosperms (Zhao et al. 2009; Chaiprasongsuk et al. 2018; Zhuge et al. 2023), a phylogenetic perspective suggests that at least one major shift in substrate preference has occurred in the IAMT enzyme lineage. In particular, the basil plant, *Ocimum basilicum* (Lamiales), encodes an IAMT ortholog that preferentially methylates phenylpropanoids, like cinnamic acid (CA) and p-coumaric acid, and exhibits no detectable activity toward IAA (Kapteyn et al. 2007) (Fig. 1). It remains unclear whether gene duplication occurred prior to this evolutionary substrate preference shift toward CA, because no additional IAMT homologs have been reported in *Ocimum* and no IAMT-like enzymes have been experimentally studied from other members of Lamiales. Furthermore, since so few other lineages related to *Ocimum* have been studied, it is possible that IAMT-like enzymes from other asterid species may also show methylation preferences for substrates other than IAA.

In this study, we investigated the phylogeny and functional characteristics of IAMT enzymes resulting from 150 million years of angiosperm evolution. Specifically, we (i) tested substrate specificity conservation among IAMT enzymes from diverse angiosperm species, (ii) assessed whether gene duplication within the IAMT clade has led to functional divergence in substrate recognition, particularly within Lamiales, and (iii) used a phylogenetic context to determine which active site residues might be most important for the evolution of CA methylation. To investigate these questions, we conducted a discovery-based classroom research project that combined comprehensive phylogenetic analyses with biochemical substrate profiling of IAMT orthologs from phylogenetically diverse plant species.

## Results and Discussion

### IAMT Orthologs Are Widespread Across Seed Plants

**Fig. 3.**
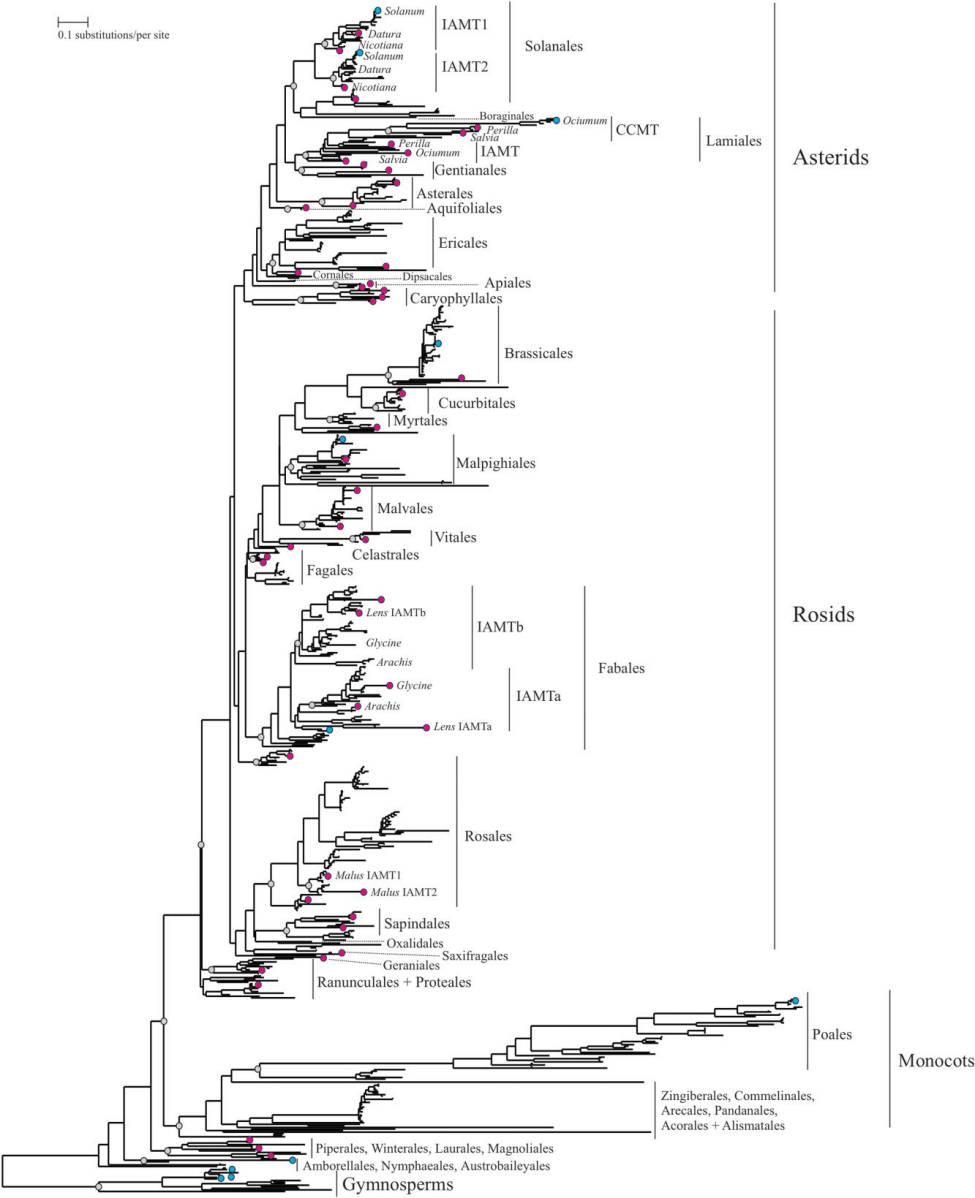
A phylogenetic analysis of IAMT sequences (log-likelihood: −70027.0912) indicates that most land plants have retained at least one IAMT ortholog. Lineages that have experienced gene duplication are labeled to differentiate the paralogs. Lineages shown with blue dots mark enzymes that have been functionally characterized previously while lineages shown with magenta dots were functionally characterized in this study. Circles at particular nodes indicate branch support values > 90.

A maximum likelihood phylogeny of 704 protein sequences reveals that all known seed plant IAMT enzymes are monophyletic, indicating that the clade originated at least 250 mya and that orthologs have been retained in nearly all descendant lineages (Fig. 3) (Zhao et al. 2007, 2008; Goto et al. 2022; Zhuge et al. 2023; Koeduka et al. 2024; Wang et al. 2024, 2025). The tree possesses protein sequences from plant species drawn from 44 orders that collectively span the major gymnosperm and angiosperm lineages, including magnoliids, monocots, rosids, and asterids, suggesting that conservation of an IAMT ortholog, presumably for auxin methylation, is an important aspect of seed plant biology. Not only have IAMT orthologs been conserved throughout seed plant history, several asterid and rosid orders encode multiple copies of IAMT. In Solanales, Lamiales, and Fabales, it appears that independent gene duplications occurred early during the evolution of the orders since anciently divergent genera appear to encode one of each duplicate (Fig. 3). One other apparent case of gene duplication in Rosales is also evident from this tree but appears to be relatively recent since the two *Malus* duplicated IAMT-type genes are very closely related (Fig. 3). For those angiosperm orders that have high-quality genomes and transcriptomes available, nearly all appear to have retained at least one IAMT ortholog. However, BLAST analyses did not recover an IAMT ortholog from Asparagales even though multiple members from both Orchidaceae and Asparagaceae have high-quality genomes available (Cai et al. 2015; Harkess et al. 2017). If IAMT is truly lacking from this order, it would represent yet another example of lineage-specific gene loss in the SABATH enzyme family. Both GAMT and SAMT were lost in Poales during early monocot evolution (Zhang et al. 2020; Dubs et al. 2022). In addition, the common ancestor of asterids also appears to have lost GAMT because no descendant species are known to encode it (Zhang et al. 2020).

### Substrate Preference Is Preserved Across IAMT Orthologs

**Fig. 4.**
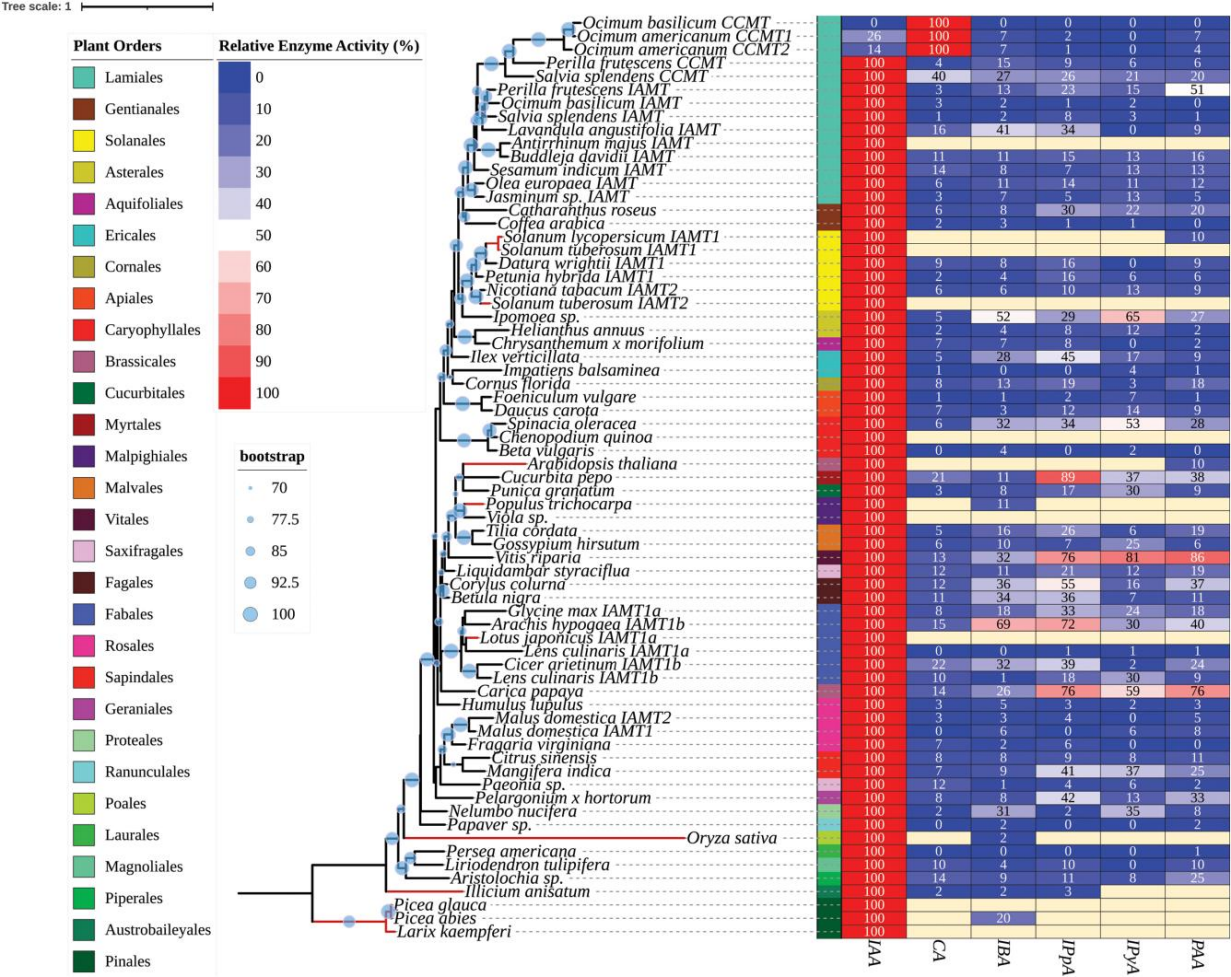
The phylogenetic tree of 69 IAMT orthologs from both gymnosperms and angiosperms shows that activity with IAA is highly conserved over seed plant history with only one recent switch in preference toward cinnamic acid detected within *Ocimum*. Maximal activity with a particular substrate was set to 100 and used to relativize activity with all other substrates for each enzyme. Red lineages designate enzymes that have been previously characterized. Empty cells mark substrates not used in enzyme assays. Circles on branches indicate levels of branch support determined with the ultrafast bootstrap.

To assess functional variation of IAMT orthologs, we performed assays of seed plant enzymes from 26 orders, 35 families, and 52 species sampled from throughout flowering plant phylogeny (Fig. 3). We used a panel of substrates including known natural auxins and/or their precursors, including IAA, phenyl acetic acid (PAA), IBA, indole-3-pyruvic acid (IPyA), and IPpA, as well as the specialized metabolite, CA (Fig. 2). Of the 59 newly reported IAMT orthologs shown in Fig. 4, all but three showed high relative activity with IAA. In order to evaluate the kinetic parameters of two of the characterized enzymes, we assayed *Liriodendron tulipifera* IAMT (LtIAMT) and *Salvia splendens* CCMT (SsCCMT). These were chosen since no IAMT-type enzyme from either the magnoliid or asterid lineage has been characterized in terms of steady-state kinetics. Both of these enzymes exhibited typical Michaelis–Menten saturation kinetics with LtIAMT showing an apparent *K_M_* for IAA of 87 µM, whereas the apparent *K_M_* of SsCCMT for IAA was 77 µM. The corresponding apparent turnover numbers (*k*_cat_) were 2.0 × 10^−4 ^S^−1^ for LtIAMT and 2.7 × 10^−4 ^S^−1^ for SsCCMT. Our *K_M_* values for IAA are comparable to estimates for IAMT from a wide array of seed plants including *P. trichocarpa*, *I. anisatum, O. sativa*, *A. thaliana*, and *Picea glauca* which range between 13 and 122 µM (Zubieta et al. 2003; Zhao et al. 2007, 2008, 2009; Koeduka et al. 2024). The high conservation of IAMT methylation preference suggests that modulation of IAA activity is crucial for basic flowering plant biology (Abbas et al. 2018). Both SAMT and GAMT enzyme substrate preferences have also been investigated across numerous angiosperm lineages and, like IAMT, a high degree of conservation was also reported (Zhang et al. 2020; Dubs et al. 2022). Nonetheless, evolutionary conservation of IAMT preference for IAA is perplexing since MeIAA is rarely reported in metabolomic surveys of plants unlike the common fragrance molecule, methyl salicylate, which is produced by SAMT. Difficulties in detecting MeIAA could result from its biosynthesis being highly restricted to particular tissues or occurring only during specific developmental stages. Also, if IAA is only produced in low abundance, it might be underreported because it is below the limit of detection of analytical instrumentation.

**Fig. 5.**
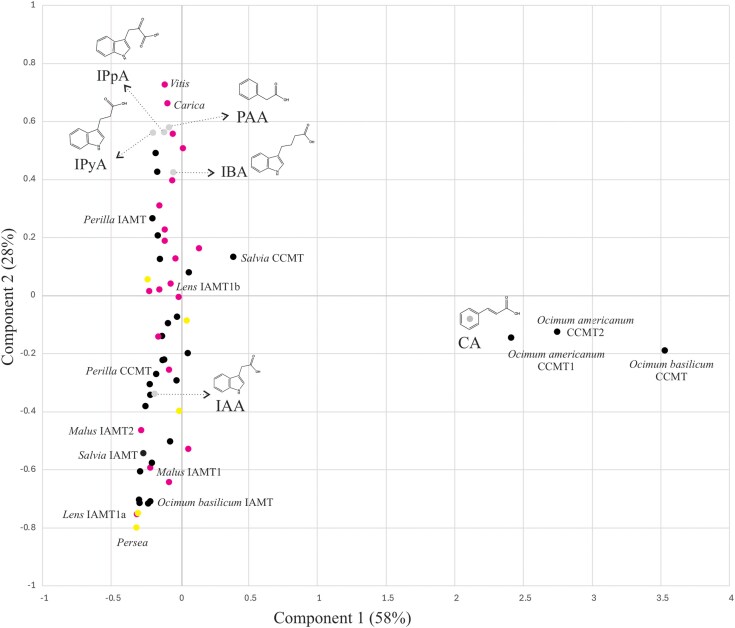
Correspondence analysis shows that IAMT-type enzymes are clearly separated by component 1 which distinguishes those that show high relative activity with CA but not other substrates. Component 2 separates the rest of the enzymes along a gradient from those that prefer IAA and show little activity with other substrates, such as that of *Persea* and *Lens* IAMT1a, to those that also prefer IAA but show higher activity with IBA, IPyA, IPpA, and PAA, such as that of *Vitis* and *Carica*. Pink dots mark the coordinates of rosid IAMT-type enzymes, black dots mark asterid-type enzymes, and yellow dots mark the positions of “other eudicot” and “magnoliid” enzymes. Labels are provided for select enzymes discussed in the text.

While many enzymes exhibit clear preference for IAA, showing only low levels of relative methylation activity with other substrates, a subset displayed relative activity greater than 50% with at least one of the other auxins (Fig. 4). These high secondary relative activities are not surprising given the structural similarities of these substrates (Fig. 2), but none was consistently preferred compared to others as the average activity with CA, IBA, IPpA, IPyA, and PAA across all enzymes was 12%, 13%, 19%, 14%, and 15%, respectively. To discern any patterns among the enzymes in terms of activities with the six substrates, a correspondence analysis was performed. The analysis revealed that IAMT orthologous enzymes are separated along the component 1 axis which clearly distinguishes enzymes that show maximal activity with CA from those that have only low relative activity with it (Fig. 5). The component 2 axis further separated the enzymes based on whether they were largely specialized for IAA methylation, such as the *Persea americana* enzyme, or whether they exhibited higher levels of relative activity with IBA, PAA, IPyA, and IPpA in addition to maximal activity with IAA, such as that of *Vitis riparia* and *Carica papaya* (Fig. 5). It remains to be seen if methylation activities with IBA, IPpA, IPyA, and PAA by IAMT are physiologically relevant in planta because the importance of the secondary activities would have to depend, in part, on in vivo concentrations. PAA levels (free or conjugated) are much lower than IAA in wild-type *A. thaliana* (Cook et al. 2016), so it is unlikely that the lower relative activities with PAA are meaningful. In the case of AtIAMT1, which showed in vitro activity with PAA that was 10% of that with IAA, no PAA methyl ester was detected in IAMT over-expressing mutant plants, suggesting limited physiological relevance in that species (Takubo et al. 2020). As for IPyA which is thought to be a direct precursor to IAA, it would likely be available for methylation in the same tissues where IAA methylation is occurring. Yet, because IPyA and IAA are present at concentrations that are within the same order of magnitude as measured by ng/fresh weight (Cooney and Nonhebel 1989; Tam and Normanly 1998), the nearly 10-fold preference for IAA would predict that mostly just MeIAA would be present in plant tissues. This suggests that if methylation of IBA, IPpA, IPyA, and PAA is important in vivo, then other paralogous SABATH enzymes may be specialized for methylating them instead of IAMT. Indeed, in *Solanum tuberosum*, a distantly related IAMT paralog known as PAAMT has recently been shown to be specialized for methylation of PAA (Wang et al. 2025).

The only IAMT orthologs that did not exhibit preference for IAA methylation are the CCMT-type paralogs from *Ocimum americanum* and *O. basilicum* (Fig. 3). Both species are known to accumulate methyl cinnamate in their tissues (Morsy and Hammad 2021). While the ObCCMT enzyme was shown previously to exhibit maximal activity with CA (Kapteyn et al. 2007), our finding of *O. americanum* enzymes that also prefer to methylate CA indicates that the preference shift might have evolved after the origin of the genus; however, it could have been much earlier. *Perilla* and *Salvia* also possess CCMT-type paralogs, but these enzymes prefer to methylate IAA (Fig. 4), so CA methylation must have originated after divergence of these three genera from their common ancestor, which is estimated at ca. 50 mya (Li et al. 2016; Rose et al. 2022). Functional analyses of CCMT-type paralogs from genera more closely related to *Ocimum* within the tribe Ocimeae, such as *Orthosiphon*, *Plectranthus*, and *Hyptis*, are required to understand the timing of the change in substrate preference (Paton et al. 2004). At the time the ObCCMT study was published in 2007, it was unclear if *O. basilicum* possesses an additional IAMT-type enzyme for IAA methylation. Our phylogenetic analysis (Fig. 3) and experimental assays (Fig. 4) demonstrate that *O. basilicum* still maintains one IAMT paralog that strongly prefers to methylate IAA. Therefore, specialization for CA methylation did not come at the expense of IAMT function in *Ocimum* species (Fig. 4). However, it remains unclear why all other Lamiales species assayed retain two copies of homologs specialized for the same substrate, IAA.

Methyl cinnamate is an important volatile in floral and fruit fragrance, but only two SABATH enzymes have been demonstrated to produce the metabolite in plants: *Ocimum* CCMT and the distantly related, paralogous CAMT enzyme from the liverwort, *Conocephalum* (Zhang et al. 2019). Our experimental assays did not find any other IAMT enzymes that have high relative activity with CA (Figs. 4 and 5), so it remains unknown how methyl cinnamate is biosynthesized in other plants. Functional studies of IAMT from species known to emit methyl cinnamate, such as *Stanhopea jenischiana* (Orchidaceae) (Williams and Whitten 1999) or *Eucalytptus olida* (Myrtaceae), could be fruitful (Smale et al. 2000). However, given that no IAMT ortholog could be found in our bioinformatic analyses for orchids, it suggests that a different SABATH enzyme, or other type of methyltransferase, must be involved in methyl cinnamate production in that lineage. Many plant metabolites, including methyl cinnamate, are produced by convergently evolved members of the SABATH family (Kapteyn et al. 2007; Zhang et al. 2019) as well as many other enzyme families (Weng et al. 2011), making this a likely possibility for orchids at the very least. Other plant families such as Rosaceae, Passifloraceae, Fabaceae, and Berberidaceae are also reported to contain at least one species that produces methyl cinnamate, thereby providing additional lineages in which to investigate the enzymatic basis for its biosynthesis (Knudsen et al. 2006).

Because it appears that enzyme substrate preference change occurred after gene duplication in *Ocimum*, we sought to investigate whether other duplicate IAMT enzymes also evolved divergent substrate preferences. As shown in Fig. 5, there is some evidence that duplication of IAMT orthologs leads to divergent substrate preferences. Specifically, three duplicated IAMT-type enzymes from the same species are separated by relative substrate preferences in the multivariate correspondence analysis. For instance, while both *Salvia* and *Perilla* of Lamiales encode an enzyme that is highly specific for IAA, they also encode a second enzyme that has higher relative activities with the other structurally similar substrates in addition to IAA (Fig. 5). The same is true for the IAMT1a and IAMT1b enzymes from *Lens* (Fig. 5). In contrast, the two *Malus* enzymes are highly similar in terms of relative substrate preferences and not separated by the multivariate analysis, which is probably a reflection of the fact that they are recently duplicated and still exhibit 95.6% sequence identity. This pattern for *Malus* is consistent with prior reports for recently duplicated Solanaceae paralogs, StIAMT1 and StIAMT2 from potato that also prefer to methylate IAA over PAA (Wang et al. 2025). While there is limited evidence for substrate preference evolution in the paralogous IAMT-type enzymes we investigated, there is published evidence for gene expression differences among them. For instance, in *Lotus japonicus*, LjIAMT1a is expressed in roots and contributes to nodule formation, whereas LjIAMT1b is predominantly expressed in shoots and leaves (Goto et al. 2022). Similarly, in *S. tuberosum*, StIAMT1 is expressed in the tuber and roots, while StIAMT2 is restricted to flowers (Wang et al. 2025). In *Solanum lycopersicum*, SlIAMT1 was expressed in germinating seeds, roots, young leaves, and fruits while SlIAMT2 was expressed in flower buds (Zhang et al. 2021). These patterns are consistent with the hypothesis that, following gene duplication, regulatory divergence may precede shifts in substrate preference.

### Sequence and Structure Determinants of IAMT and CCMT Substrate Preferences in Lamiales

**Fig. 6.**
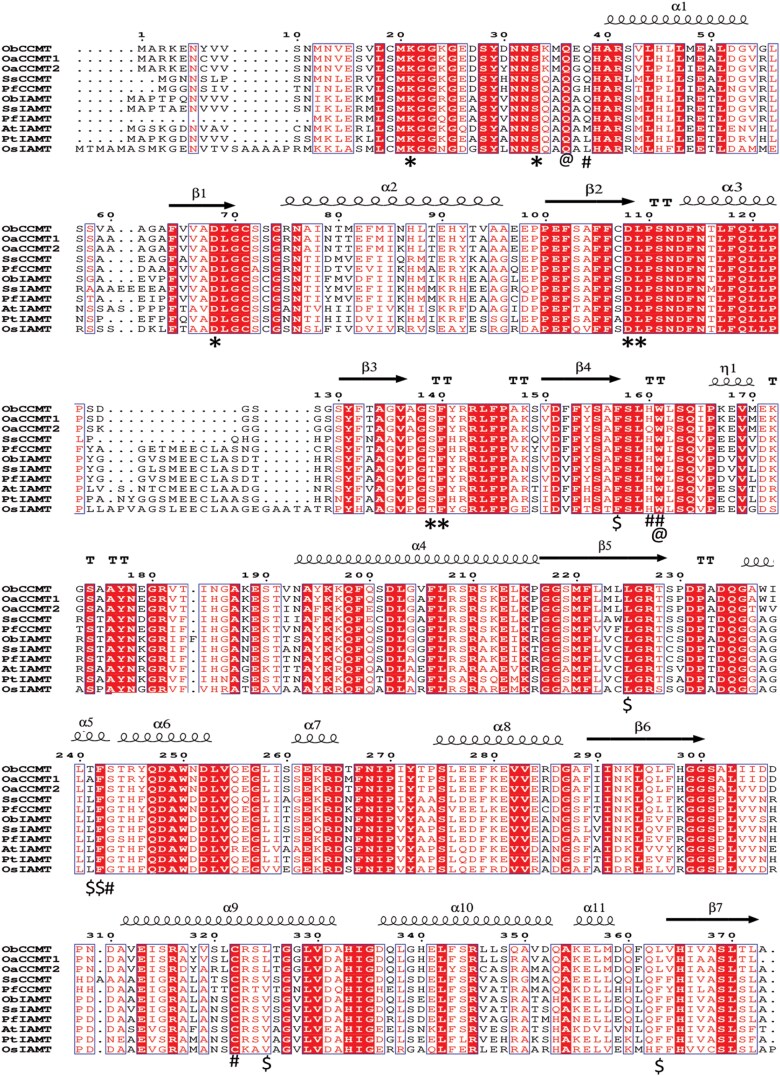
Amino acid alignment of IAMT and CCMT enzymes. The alpha helix (shown as helices) and beta pleated sheet (shown as arrows) secondary structural elements in addition to turns (shown as “T”) are placed above the alignment and were reported from the crystal structure of AtIAMT (PDB code: 3B5I). Residues interacting with SAM/SAH are indicated with “*”, carboxyl group interacting residues with “@”, aromatic moiety interacting residues with “$”, and residues that are predicted to bind with CA are indicated with “#”.

**Fig. 7.**
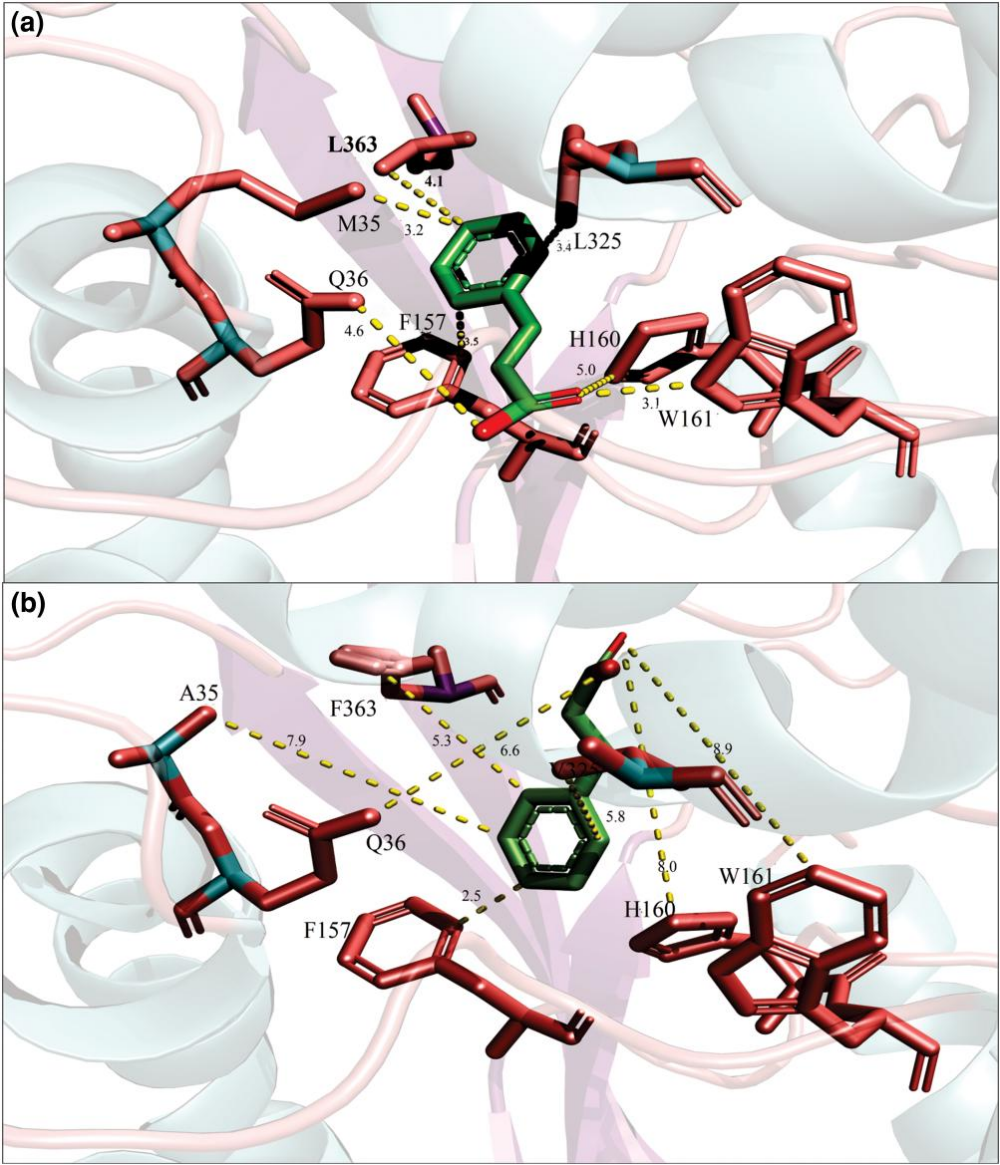
Molecular docking of wild-type ObCCMT and the IAMT-like triple mutant ObCCMT with CA. a) ObCCMT and CA docking shows the pocket clamp formed by M35, L325, and L363 packing against CA at van der Waals distances (3.5 Å, 3.4 Å, and 4.1 Å, respectively), while nearby positioning residues (Q36 4.6 Å; H160 5.0 Å; F157 3.5 Å) maintain the catalytic pose of the acid. b) Docking of the mutant ObCCMT (M35A/L325V/L363F) with CA shows that replacing the clamp with IAMT-like side chains results in greater distances from the substrate: A35 at 7.9 Å; V325 at 5.8 Å; and F363 at 5.3 Å. Concomitant increases in distance from the substrate are also observed for the positioning residues with Q36 at 16.6 Å; H160 at 8.0 Å; W161 at 8.9 Å; and F157 at 2.5 Å. Dashed yellow lines indicate measured distances (Å).

To understand the amino acid positions involved in the substrate preference switch to CA in *Ocimum*, we compared active site residues among closely related Lamiaceae IAMT enzymes, their CCMT paralogs, and previously characterized sequences (Kapteyn et al. 2007; Zhao et al. 2008). Notably, key residues in SAM/SAH binding and catalysis (K21, S33, D68, D108, L109, and F140) are strictly conserved among all sequences (Fig. 6) (Zubieta et al. 2003; Zhao et al. 2007, 2008). This conservation was previously noted in IAMTs and SAMTs and explains the consistency of methyl donor preference among SABATH enzymes (Zhao et al. 2008). In addition, residues involved in forming hydrogen bonds to the carboxylate moiety of IAA/CA (K21, Q36, W161) are identical in all sequences and are conserved in other members of the SABATH family. For instance, in *Clarkia breweri* SAMT (CbSAMT), the homologous K10, Q25, and W151 fix salicylic acid at ∼4.35 Å from the sulfonium center of SAH (Zubieta et al. 2003; Wang et al. 2024). Similarly, given that Q38, H162 and W163 also play a carboxylate-positioning role in *Catharanthus roseus* LAMT (CrLAMT) (Petronikolou et al. 2018), it indicates that substrate preference change in *Ocimum* CCMT enzymes probably does not stem from differences in carboxylate positioning or SAM binding. Instead, CCMT in *Ocimum* exhibits unique substitutions at several conserved positions. For instance, S243 in ObCCMT is equivalent to glycine in AtIAMT and was previously hypothesized to enlarge and polarize the binding pocket (Fig. 6) (Kapteyn et al. 2007). In addition, *Ocimum* CCMTs lack an 11-residue segment present in AtIAMT within the α3/β4 loop (alignment positions 125 to 136) that has been reported as a non-catalytic core segment (Kapteyn et al. 2007). In our alignment (Fig. 6), SsCCMT also possesses the 11-residue loop deletion but still prefers to methylate IAA therefore arguing against this structural mutation being responsible for CA methylation preference. Our alignment and docking models identify three potentially important differences between ObCCMT and IAMT enzymes at alignment positions 35, 325, and 363 (Figs. 6 and 7). In ObCCMT, the IAMT residues A35, V325, and F363 were replaced by Met, Leu, and Leu, respectively. These substitutions provide favorable contacts for CA as M35 provides a large, polarizable surface at the pocket mouth that can accommodate the flat phenyl ring of CA; L325 shapes a shallower hydrophobic cavity that fits the smaller aromatic ring of CA; and L363 removes an indole-biased π face, allowing CA to dock close to the sulfonium (Fig. 7). To investigate the roles of these residues for interactions with CA, we docked the substrate into an ObCCMT triple mutant (M35A/L325V/L363F). Docking shows that in wild-type ObCCMT (WTObCCMT), Q36 and W161 hydrogen bond to the carboxylate moiety in the required orientation for methyl transfer. In contrast, CA is no longer fixed in proximity to these residues in the best catalytic pose of the mutant (Fig. 7). In WTObCCMT, M35, L325, and L363 sit 3.2 Å, 3.4 Å, and 4.1 Å, from CA, respectively (Fig. 7a). After the mutations, the corresponding side chains are displaced beyond contact range (A35 at 7.9 Å, V325 at 5.8 Å, and F363 at 5.3 Å; Fig. 7b). Concomitantly, key carboxylate clamp residues such as Q36 and W161 become more distant by 2 Å and 6 Å, respectively.

**Fig. 8.**
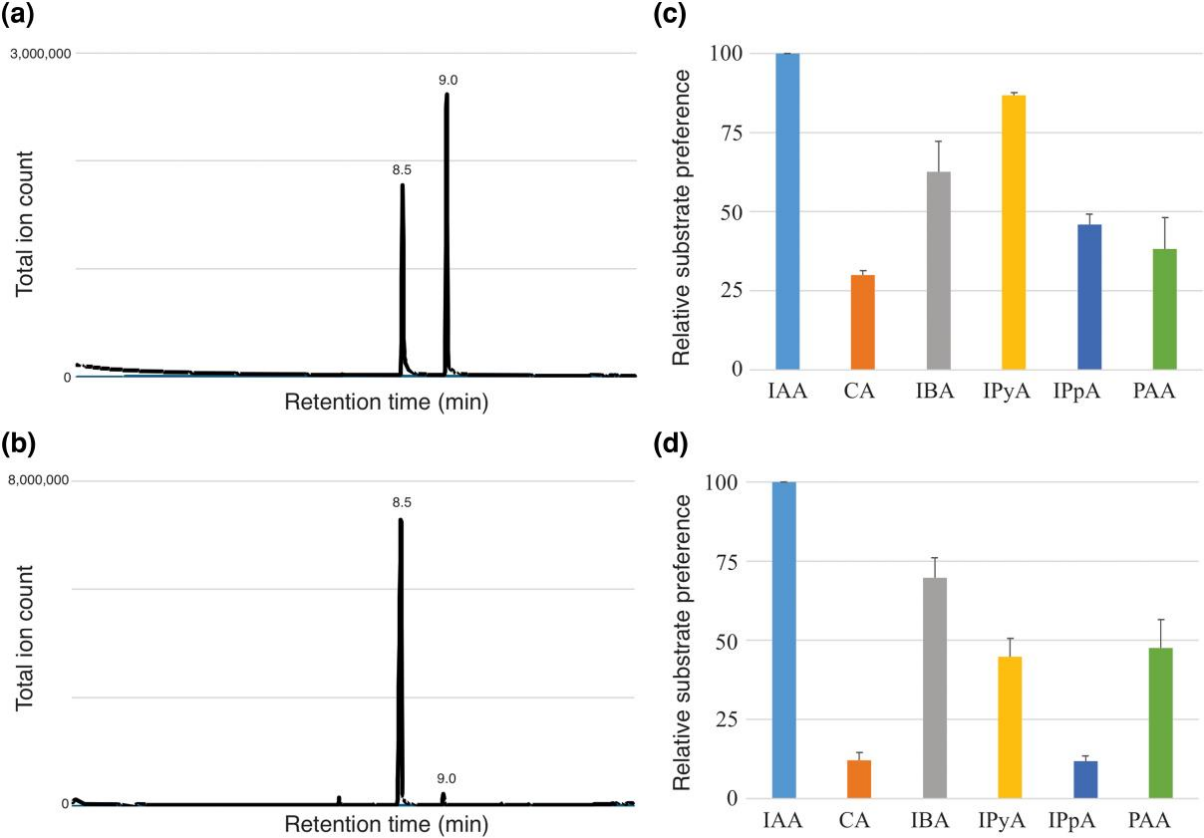
Mutations at three sites amino acid positions do not change substrate preference of CCMT-type enzymes. a) GC-MS chromatogram showing that wild-type ObCCMT methylates CA to form MeCA which is detected at 9.0 min. The peak at 8.5 is indole which is produced as a catabolite in E. coli cultures. b) GC-MS chromatogram showing that the M35A, L325V, and L363F triple mutant, ΔObCCMT, still is capable of producing MeCA, though apparently less than wild type. c) The ancestral enzyme, Node 5, that gave rise to CA-preferring CCMT enzymes in *Ocimum* prefers to methylate IAA but shows high relative activity with the other substrates tested. d) Introduction of the three mutations, A35M, V325L, and F363L, into the ancestral Node 5 enzyme, ΔNode 5, did not increase relative activity with CA as was predicted.

Because the computational analyses suggested that M35A/L325V/L363F would result in the loss of CA methylation, we sought to experimentally test that prediction in two ways. The first approach was an evolutionary reversal, whereby we introduced M35A/L325V/L363F into ObCCMT (ΔObCCMT) with the expectation that it would result in loss of activity with CA. To our surprise, neither crude extracts nor purified ΔObCCMT displayed detectable activity with CA or any other substrate in our radioactive assays. The primary reason appears to be that very little soluble protein is produced as it is mostly found in the cell pellet likely sequestered within inclusion bodies (Fig. S1). Therefore, we chose to assay the triple mutant using an in vivo *E. coli* assay in which we supplement the LB liquid medium with CA and IAA and then use gas chromatography–mass spectrometry (GC-MS) to detect methylated products, which has been shown to allow for assays of other insoluble SABATH proteins as performed in this and prior studies (Dubs et al. 2022). As is shown in Fig. 8, ΔObCCMT produced a peak corresponding to MeCA, although it is substantially less than wild-type ObCCMT. Thus, it appears that reverse mutations of these three amino acid positions did not alter substrate preference. While it is tempting to conclude that the enzyme is less efficient at methylating CA, this assay does not control for amount of properly folded functional protein. Neither the wild-type nor mutant ObCCMT produced detectable amounts of MeIAA.

The second approach was to resurrect the ancestral CCMT that gave rise to the *Ocimum* CCMT enzymes (Fig. S2) and make a forward evolutionary mutant to evaluate the impact of the three mutations on the gain of activity with CA. In contrast to WTObCCMT, which is highly specialized for CA methylation, the ancestral CCMT enzyme at Node 5 exhibited IAA methylation preference, while also retaining substantial activity toward other substrates, including IBA (62%), IPpA (46%), IPyA (87%), PAA (38%), and CA (30%) (Fig. 8). This finding reinforces the notion that the gain of CA methylation preference is recent and perhaps restricted to the *Ocimum* lineage. We subsequently introduced three amino acid replacements A35M/V325L/F363L that we predicted would result in a switch to CA methylation preference. This mutant, ΔNode 5, did not demonstrate enhanced CA methylation activity and still preferred to methylate IAA. Thus it appears that M35, L325, and L363 were not important for the evolution of CA methylation preference in aggregate; therefore, it seems likely that different sites were crucial for changing substrate preference. It is also possible that M35, L325, and L363 were important for the evolution of CA methylation activity, but there are strong epistatic effects of additional mutations at other sites. There are a total of 60 amino acid changes that have occurred along the branch linking Node 5 to the *Ocimum* CA-preferring enzymes. We propose that future studies should investigate the impact of Q34K and V271I because those substitutions appear to be restricted to CA-preferring enzymes and the homologous sites have been shown to impact the evolution of substrate preference in other SABATH enzymes (O'Donnell et al. 2021; Catania et al. 2024). Additionally, G243S should also be investigated since our docking studies suggest the residue may bind to CA.

## Conclusion

The results of our phylogeny-guided experimental study show a clear pattern: IAMT enzymes show a conserved preference to methylate IAA over structurally similar substrates. This preference is likely a property of most seed plant IAMTs despite the fact that the product of the enzymatic reaction, IAA methyl ester, has rarely been reported from plant metabolomes. Yet, like other specialized metabolite-producing enzyme families such as the BAHD acyltransferases (Kruse et al. 2022) and terpene synthases (Chen et al. 2011), SABATH family members show a remarkable evolutionary flexibility to diverge and acquire novel substrate preferences after gene duplication. The mechanistic basis for substrate preference shifts in other SABATH enzymes has been shown to involve very few amino acid replacements without much evidence for strong epistatic interactions (Huang et al. 2016; O'Donnell et al. 2021; Catania et al. 2024). While a simple mutational basis for enzymatic divergence has also been shown in other specialized metabolic enzymes (Fan et al. 2016), the mechanistic basis for the evolution of CA methylation preference in CCMT remains unclear. In cases where sequence divergence is high, as it is for *Ocimum* CCMT, increased sampling of closely related sequences could help pinpoint which amino acid replacements were concomitant with substrate preference switches. In addition, mutagenesis of combinations of blocks of mutations rather than only one or few mutations will likely be required to understand the extent to which epistasis permitted or limited the evolution of substrate preference switches.

## Materials and Methods

### Collaborative Classroom Research Framework

This IAMT enzyme evolution project was implemented as a semester-long, upper-level course-based undergraduate research experience (CURE) over three consecutive years. The collaborative course investigated enzyme substrate preference evolution which required students to apply numerous molecular techniques for each species studied including RNA extraction, cDNA synthesis via RT-PCR, cloning gene sequences into expression vectors, and heterologous expression in *E. coli*, followed by GC-MS to assess product formation. Experimental procedures were complemented with computational analyses that included multiple sequence alignment and phylogenetic analysis to explore evolutionary relationships among IAMT homologs and make protein functional predictions. Throughout the semester, detailed lab notebooks documented experimental procedures and results. Altogether, these exercises not only provided foundational training in molecular biology and biochemistry but also generated original data to test hypotheses about enzyme evolution. Nearly all the sequences cloned and characterized in this study originated from the course, and the findings directly informed the functional and phylogenetic insights presented. This CURE course continues to serve as both a teaching and research platform that has promoted a deeper understanding of plant enzymatic evolution (see also Dubs et al. 2022). Although the CURE provides an authentic research experience and has pedagogical value, there are clearly limitations to what can be achieved in a single semester. Due to time constraints, it is difficult to achieve sufficient replication and accommodate every aspect of a robust experimental design. Nonetheless, the course we have implemented provides a meaningful exposure to the scientific method and molecular biology techniques and achieves a broad sampling of enzymes from a wide array of species.

### Species Sampling and IAMT cDNA Synthesis

In order to evaluate how conserved IAMT substrate preference is throughout angiosperms, species were sampled from as many flowering plant orders as possible. The origin of CA methylation in *Ocimum* was investigated by sampling closely related species within the Lamiales. In all cases, species were only sampled for molecular analysis if bioinformatic analyses indicated that candidate IAMT gene sequences were available for primer design. All plant material was collected from the Finch Greenhouse and surrounding campus grounds at Western Michigan University. Total RNA was extracted using the Spectrum Plant Total RNA Kit (Sigma–Aldrich), following the manufacturer's instructions. Approximately 0.1 g of fresh tissue per sample was immediately frozen in liquid nitrogen and finely ground using a sterile plastic pestle. RNA was eluted in 50 µL of elution solution and stored at −80 °C until use. RNA integrity was assessed via 1.5% agarose gel electrophoresis and quantified using a NanoDrop spectrophotometer (Thermo Fisher Scientific). Primers were designed using Primer3Plus from predicted sequences obtained from BLAST analyses and were synthesized by Integrated DNA Technologies (Table S1). Complementary DNA (cDNA) was synthesized and amplified using the SuperScript IV One-Step RT-PCR System with Platinum SuperFi DNA Polymerase (Invitrogen, Carlsbad, CA). Each 50 µL RT-PCR reaction included 2.5 µL of both forward and reverse primers (10 µM), 25 µL of 2× reaction mix, 0.5 µL of SuperScript IV Platinum Taq enzyme mix, and approximately 500 ng of total RNA as template. RNase-free water was added to adjust the final reaction volume to 50 µL. Thermocycling was carried out with the following conditions: reverse transcription at 50 °C for 10 min with RT inactivation of 98 °C for 2 min, followed by 40 cycles of PCR with denaturation at 98 °C for 10 s, annealing at 50 °C for 10 s, and extension at 72 °C for 45 s. A final extension was performed at 72 °C for 5 min.

### Cloning and Transformation

To obtain pure cDNA fragments for cloning, gel purification was performed using the GeneJet Gel extraction kit (Thermo Fisher Scientific). Amplified cDNA products from 48 species were cloned into either the pTrcHIS, pTrcHIS2, or pBAD expression vectors using the TOPO TA Cloning Kits (Invitrogen, Carlsbad, CA). In addition, genes from seven species were synthesized and subcloned into either pET15b (Genscript Corp) or pET28a (Twist Bioscience, CA). Table S2 lists the vector used for each cloned gene from each species studied. Prior to TA cloning, adenylation of cDNA was carried out using a 1:1 ratio of cDNA and 2X Taq Master Mix (Invitrogen, Carlsbad, CA) that was incubated at 72 °C for 20 min. Ligation reactions and transformations into chemically competent TOP10 *E. coli* cells were performed following the manufacturer's instructions. Approximately 100 µL of transformed cells was plated on LB agar supplemented with 100 µg/mL ampicillin or 50 µg/mL kanamycin and incubated overnight at 37 °C. Resulting colonies were screened for correct insert size by colony PCR using vector-specific forward and reverse primers: either pTrcHISF+R or pBADF+R. Each 25 µL PCR reaction contained 0.5 µL of each primer (10 µM), 12.5 µL of 2× Taq Master Mix (Invitrogen, Carlsbad, CA), and molecular-grade water. A small amount of bacterial cells (∼0.5 µL) was transferred into the reaction mix using a sterile pipette tip. PCR products of the expected size were purified using the GeneJet PCR Purification Kit (Invitrogen, Carlsbad, CA) and submitted to Genewiz (South Plainfield, NJ) for Sanger sequencing to confirm sequence identity and insert orientation. cDNA accession numbers can be found in Table S2.

### Recombinant Protein Expression, Enzyme Activity Assays, and GC-MS Analysis

For protein expression, single colonies containing sense-orientation constructs were grown overnight in 5 mL of LB medium containing 100 µg/mL ampicillin or 50 µg/mL kanamycin at 37 °C with shaking. The next day, 48 mL of fresh LB-antibiotic medium was inoculated with 2 mL of the overnight culture and incubated at 37 °C until cultures reached an OD_600_ of ∼0.6. Protein expression was induced by adding a final concentration of 1 mM IPTG for bacteria transformed with pTrcHIS, pTrcHIS2, pET28a, and pET15b vectors or 0.2% final concentration of arabinose for those transformed with the pBAD plasmid. Enzyme assays were simultaneously initiated using a modified version of a GC-MS-based methylation assay in which equimolar concentrations of IAA and CA were added to each culture at a final concentration of 1.0 mM to assess relative substrate preference (Barkman et al. 2007; Dubs et al. 2022). After protein induction and substrate addition, cultures were incubated overnight at room temperature with gentle shaking to allow methylation reactions to occur in vivo. After 12 to 16 h, cultures were centrifuged at 4,000 × *g* for 20 min at 4 °C to pellet the bacterial cells. The supernatants were collected and extracted with 5 mL of hexane to isolate volatile methylated products. One microliter of extracted products was analyzed via GC-MS using an HP6890 GC System equipped with a DB-5 capillary column and an HP5973 Mass Selective Detector. The oven was programmed to hold at 40 °C for 2 min, followed by a temperature ramp of 20 °C/min to 300 °C with a final 2-min hold. Relative enzymatic activity was quantified by integrating chromatographic peaks corresponding to methylated products. Negative controls consisted of cultures transformed with antisense vectors processed under identical conditions. Three IAMT enzymes from *Viola*, *Chenopodium*, and *Antirrhinum* were tested only with IAA and CA using the GC/MS assay described above due to enzymes being insoluble.

### Post-classroom Relative Activity Assays

Due to time constraints imposed by a single semester CURE, complete enzymatic studies were not possible. Therefore, following the completion of the classroom component, previously generated bacterial glycerol stocks transformed with IAMT homologs were used for further enzyme analysis. Glycerol stocks were used for protein expression as described above and after overnight induction at room temperature, cells were harvested by centrifugation at 4,000 × *g* for 20 min at 4 °C. Supernatants were discarded, and the resulting cell pellets were resuspended in 3.6 mL of enzyme wash buffer (50 mM sodium phosphate dibasic, 300 mM NaCl, 10 mM imidazole, and 12% v/v glycerol). Cells were lysed via sonication using five cycles of 10 s of disruption followed by 30 s of rest on ice. The lysates were then centrifuged at 4,000 × *g* for 20 min at 4 °C, and the supernatant was collected as crude enzyme extract. All His-tagged proteins were subsequently purified using the TALON spin column protein purification kit (Takara Bio, CA), following the manufacturer's protocol. To preserve enzymatic activity, 60 µL of 0.1 M dithiothreitol (DTT) was added to the purified samples prior to storage at −80 °C.

Enzymatic activity of purified IAMT enzymes was assayed using the MTase-Glo Methyltransferase Assay (Promega, WI), which detects S-adenosylhomocysteine (SAH) as a byproduct of methyltransferase reactions and has been shown to be effective for SABATH enzyme assays (Fallon et al. 2024). In methyltransferase reactions, SAM is converted to SAH by donating a methyl group to substrate and MTase-Glo reagent hydrolyzes the SAH, yielding adenosine and L-homocysteine (Loenen 2006). The adenosine is then phosphorylated to ultimately form ATP which then is detected by a luminescence signal (Hsiao et al. 2016). Enzymatic assays were performed using equimolar concentrations of IAA, CA, IBA, IPyA, IPpA, or PAA (Fig. 2). These compounds were selected for several reasons. First, all but CA have structural similarities to IAA and therefore may be recognized as substrates. Second, IPyA is a biosynthetic precursor to IAA (Mashiguchi et al. 2011; Cook et al. 2016). Third, one or more of the substrates were assayed using IAMT in previously published studies (Zhao et al. 2008; Takubo et al. 2020; Wang et al. 2025). Each 22 µL enzymatic reaction included 5 µL of 4× reaction buffer (250 mM Tris-HCl pH 7.0, 1 M NaCl, 0.5 M EDTA, 1 M MgCl_2_, 10 mg/mL BSA, and 0.1 M DTT), 2.5 µL of freshly diluted 5× MTase-Glo reagent, 0.5 µL of 1 mM MTase-Glo SAM, 0.5 µL of 50 mM substrate dissolved in 70% ethanol, approximately 20 µg/mL of purified protein, and nuclease-free water to reach the final volume. Reactions were incubated in the dark at room temperature for 15 min, followed by the addition of 10 µL of MTase-Glo detection solution and an additional 15-min incubation under the same conditions. Luminescence was then measured using a Spark Multimode Microplate Reader with no attenuation and 1,000 ms integration time. Negative controls contained 0.5 µL of 70% ethanol in place of the substrate and were included for each enzyme to account for background signal and ensure specificity of the assay. After background subtraction, the highest activity with a particular substrate was set to 100%. Enzyme relative activities with other substrates were then scaled to 100% to provide an intuitive measure of the level of activity with each substrate relative to one another. Each enzymatic assay was performed in triplicate.

During the first semester of this investigation, 14 genes were cloned without a His-fusion tag to produce native protein because of reports that the 6x-His tag can potentially affect SABATH enzyme activity (Mizuno et al. 2003). For those enzymes lacking a His-tag (Table S2), the MTase-Glo assays could not be performed. Therefore, relative substrate preference assays were performed in 50 µL reactions containing 10 µL of crude enzyme extract (25% v/v), 62.5 mM Tris-HCl buffer (pH 7.0), 625 μM substrate, and [^14^C]-SAM at 4 µM final concentration, with molecular-grade water added to volume. Following a 1-h incubation at room temperature, 250 µL of HPLC-grade ethyl acetate was added to extract methylated products. The mixtures were centrifuged at 12,000 rpm for 1 min to separate the organic phase. From this, 150 µL of the ethyl acetate layer was transferred to a scintillation vial containing 3 mL of scintillation cocktail. Radioactive incorporation was measured using a Perkin–Elmer Tri-Carb 2910 TR scintillation counter, and activity was expressed in disintegrations per minute. Each reaction was performed in duplicate. Negative control reactions were conducted using ethanol as described above. We routinely test crude enzymes for activity using this radioactive assay and have verified that it provides quantitatively similar relative substrate preference results to the MTase-Glo assay.

In order to demonstrate that the two different assays are largely comparable, we have generated radioactive assays for five enzymes that we already had MTase-Glo data for. Each enzyme was assayed against the same panel of substrates under identical reaction conditions. Using GraphPad Prism 10, we found that a Wilcoxon matched pairs signed rank test indicated no significant differences between the paired measurements (*P* = 0.062). Correlation analysis revealed a strong monotonic relationship between the two assays (Spearman *r* = 1.00, *P* = 0.016), indicating consistent ranking of substrate preferences. Bland–Altman analysis showed a mean bias of 2.86 with 95% limits of agreement ranging from −1.92 to 7.64, suggesting close agreement between the two methods. We also note that in a correspondence analysis containing paired assays for the five species, they are largely localized to a similar quadrant of the plot (Fig. S3). Finally, we also investigated the original correspondence analysis of Fig. 5 and found that enzymes interrogated with different assays did not cluster to the exclusion of others suggesting that there is not a systematic bias (Fig. S4).

### Enzyme Kinetics Analyses

To determine the Michaelis–Menten kinetic parameters, radioactive enzyme assays were performed for IAMT from *L. tulipifera* (LtIAMT) and CCMT from *S. splendens* (SsCCMT). For each enzyme, we first determined optimal conditions to ensure linear reaction velocity with respect to purified protein concentration and time. Reactions were carried out in fixed assay volumes under initial rate conditions using varying concentrations of IAA (10 to 800 μM) while [14C]-SAM was supplied at saturating concentrations (320 μM). Reactions were terminated after incubation, and product formation was quantified by measuring incorporated radioactivity. Velocities were calculated from replicate measurements and used to estimate Michaelis–Menten parameters by nonlinear regression analysis in KaleidaGraph.

### Phylogenetic Analyses

The IAMT phylogenetic tree of Fig. 3 was generated using amino acid sequences obtained by BlastP searches in the non-redundant protein sequence (nr) and Transcriptome Shotgun Assembly proteins (tsa-nr) databases of NCBI and from the OneKP database of China National Gene Bank. In addition, Core nucleotide (Core-nt), Whole Genome Shotgun contigs (wgs), and Transcriptome Shotgun Assembly (TSA) databases were used to collect additional available sequences using TBlastN searches. In all searches, the IAMT protein from *A. thaliana* was used as the query sequence (NP_200336.1). In total, 704 IAMT sequences were aligned using MAFFT software with default parameters (Katoh et al. 2019). The best model of amino acid substitution was determined by ModelFinder to be JTT+R8 (Kalyaanamoorthy et al. 2017), which was implemented for phylogenetic tree estimation by IQtree using maximum likelihood. The tree was rooted with gymnosperms since they are recovered as the sister group to angiosperm IAMT sequences as reported in Chaiprasongsuk et al. (2018) and Zhuge et al. (2023) and shown in Fig. 1. Branch support was determined using the ultrafast bootstrap with 1000 replicates (Hoang et al. 2018; Wong et al. 2025). In order to show the phylogenetic patterns of substrate preference for the 69 protein sequences shown in Fig. 4, we pruned all sequences from Fig. 3 that do not have associated experimentally determined enzymatic activity. The resulting phylogenetic tree was visualized and annotated using iTol (Letunic and Bork 2024).

For ancestral sequence reconstruction, CCMT and IAMT sequences from the Mentheae, Ocimeae, and Elsholtzieae tribes (Lamiaceae) were collected and aligned using MAFFT software using L-INS-I strategy and BLOSUM62 (Katoh et al. 2019). Next, a maximum likelihood phylogeny was inferred assuming the GTR2+FO amino acid substitution model which was determined to be the best model by ModelFinder (Kalyaanamoorthy et al. 2017). IQtree was then used to estimate the ancestral CCMT sequence corresponding to Node 5 (Fig. S2). The average site-specific posterior probability of the estimated amino acid sequence is 0.98 (Fig. S2). The ancestral sequence was synthesized with codon optimization for expression in *E. coli* and subcloned into pET28a, heterologously expressed, purified, and assayed using MTase-Glo following the same protocol as described above.

### Enzyme Substrate Correspondence Analysis

Principal correspondence analysis of enzyme substrate association was performed using simple correspondence analysis in Minitab Statistical Software on a contingency table whose rows were enzymes and columns were relative activities with six substrates (IAA, CA, IBA, IPpA, IPyA, and PAA). We only included enzymes shown in Fig. 4 for which all six substrates were assayed because missing data could not be accommodated by the analysis. A symmetric plot of rows and columns was generated in Minitab and further edited for visualization by CorelDraw.

### Protein Structure Modeling and Active Site Residue Analysis

To compare conserved active site residues among closely related Lamiaceae CCMT and IAMT enzymes, full length amino acid sequences of sCCMT and IAMT (ObCCMT and ObIAMT), *Perilla frutescens* CCMT and IAMT (PfCCMT and PfIAMT), and *S. splendens* CCMT and IAMT (SsCCMT and SsIAMT) along with previously studied amino acid sequences were aligned with Clustal Omega using default parameters (Madeira et al. 2024). The multiple sequence alignment was formatted with ESPript to display secondary structure and residue conservation using AtIAMT (PDB code: 3B5I) for residue numbering and mapping positions across orthologs/paralogs (Gouet et al. 1999). We modeled wild-type ObCCMT (WTObCCMT) and mutated ObCCMT (ΔObCCMT) using AlphaFold3 (Abramson et al. 2024). The modeled WTObCCMT was aligned with AtIAMT monomer and ΔObCCMT models were aligned with WTObCCMT using the alignment plugin in PyMol version 3.1.3.1 (Schrodinger 2015). Models with the lowest RMSD were chosen to continue the active site residue analysis. The CA structure was downloaded from PubChem and both ligand and receptors were converted to a PDBQT file using PyRx V 0.8; receptors were kept rigid and ligands flexible (O'Boyle et al. 2011; Dallakyan and Olson 2015). Geometries of CA were minimized using the MMFF94 option available in PyRx/OpenBabel (O'Boyle et al. 2011; Dallakyan and Olson 2015). Docking was performed with AutoDock Vina as bundled in PyRX using an orthogonal grid box centered on active site residues K21, M35, Q36, Q38, F157, H160, W161, L225, T241, F242, C322, L325, and L363 (Trott and Olson 2010; Dallakyan and Olson 2015; Eberhardt et al. 2021). AutoDock Vina parameters were exhaustiveness = 8, num_modes = 9, and energy_range = 3 kcal.mol^−1^ (Dallakyan and Olson 2015). Docked complexes were visualized in PyMol and used for measuring ligand-residue distances (Schrodinger 2015). In order to evaluate the experimental impact of the mutations, both WTObCCMT and triple mutant ΔObCCMT (M35A/L325V/L363F) were synthesized and subcloned into pET28a (Twist Bioscience, CA). These two enzymes were expressed and extracted following the methods discussed above, and enzyme activities were measured with all the six substrates using the MTase-Glo Methyltransferase Assay.

## Supplementary Material

evag167_Supplementary_Data

## Data Availability

The data matrix and complete tree with branch support shown in Fig. 3 is available at https://doi.org/10.6084/m9.figshare.31839217. The data matrix and tree used to estimate ancestral CCMT sequences shown in Fig. S2 is available at https://doi.org/10.6084/m9.figshare.30648848.
