# Supplementary material for: Conservation of IAMT Preference for Indole Acetic Acid Methylation Across 250 Million Years of Seed Plant Divergence, With Only One Recent Evolutionary Switch in Ocimum
Source: Genome Biol Evol. 2026 Jul 2;18(7):evag167. doi: 10.1093/gbe/evag167 (PMC13376665; doi:10.1093/gbe/evag167)
Supplement: evag167_Supplementary_Data [file evag167_supplementary_data.pdf]

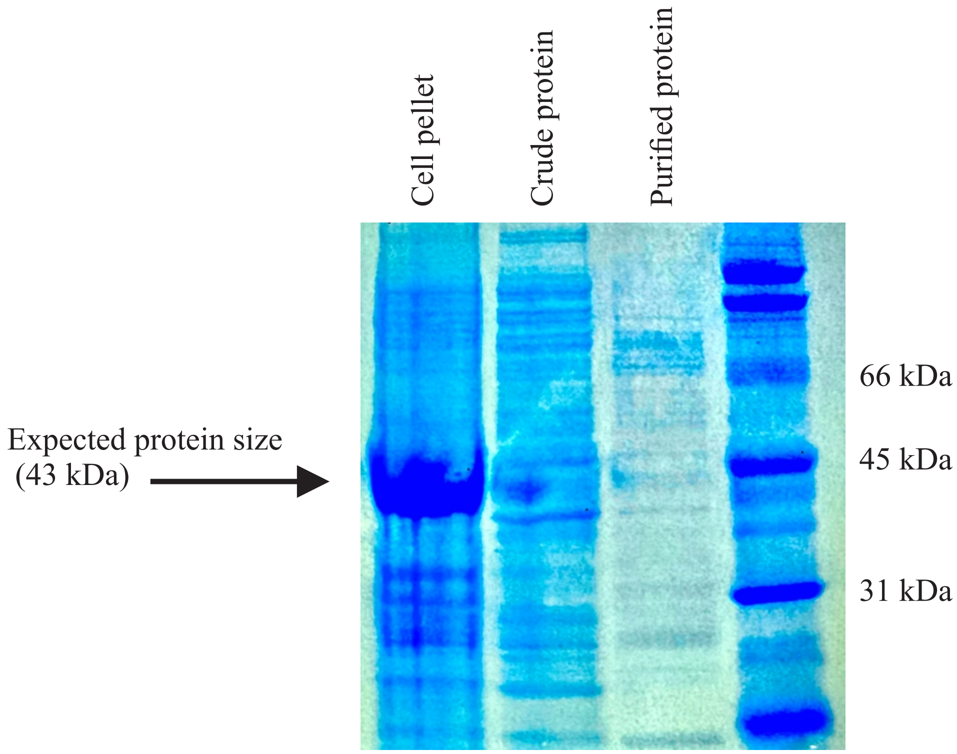

Supplementary Figure 1. SDS-PAGE gel stained with Coomassie blue showing that the ObCCMT triple mutant recombinant protein is localized to the cell pellet and is not soluble in the crude extract and therefore is not recovered in the purified fraction.

A.

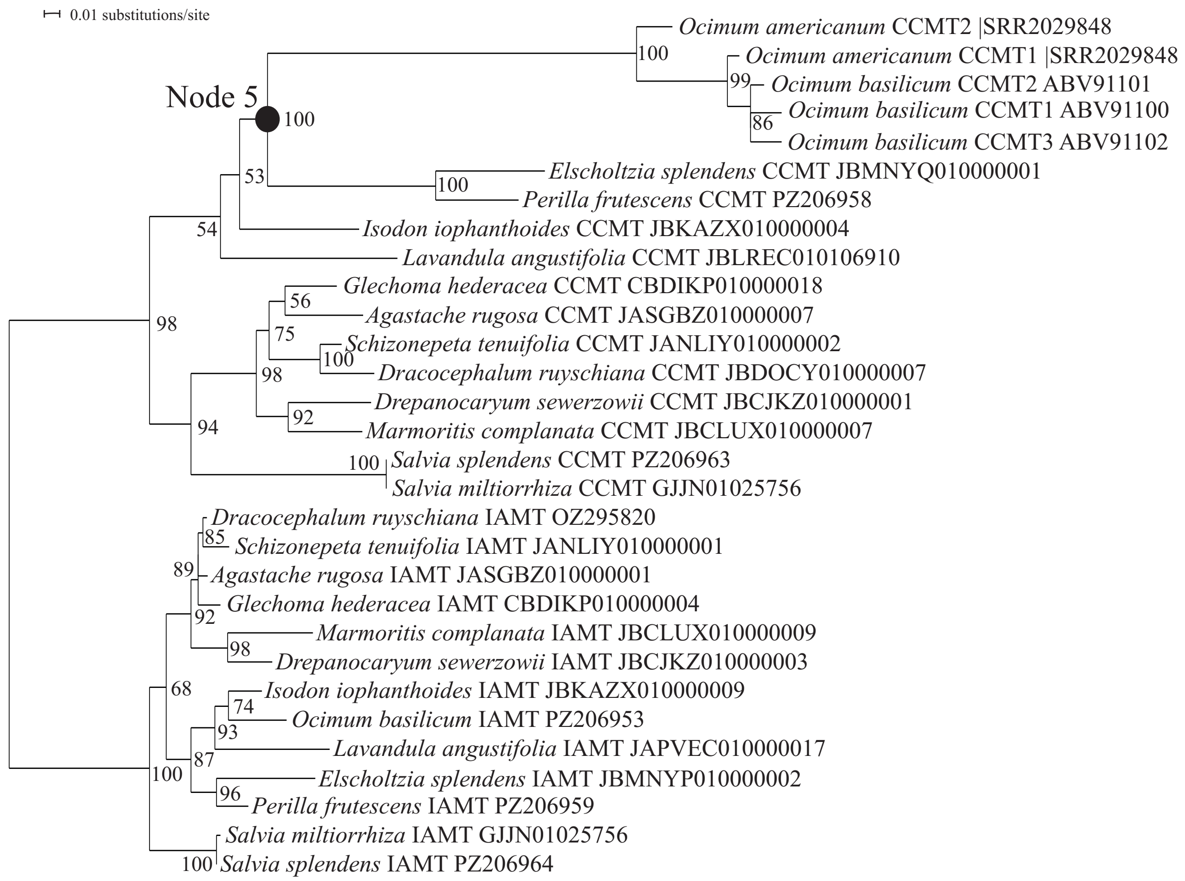

B.

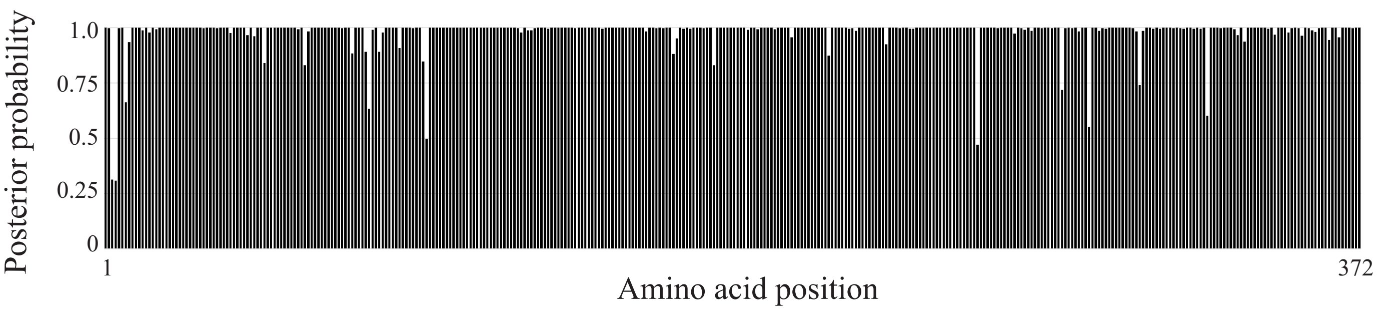

C.

MGPSENSVVSNMNLERVLSMKGGKGEDSYHNNSQAQGQHARSVLPLLIEALDGVRLSSAAGAF
VVADLGCSSGRNTINTVEVMIKHMTERYKAAAQEPPEFSAFFCDLPSNDFNTLFQLLPPYAGGGRS
YFTAGVPGSFHRRLFPAKYVDFFYSAFSLHWLSQVPEEVVDKRSTAYNEGRVFIHGAKESTVNAF
KKQFESDLGGFLRSRSKELKSGGSMFLVFLGRTSSDPTDQGGAGLLFGTHYQDAWNDLVQEGLIS
SEKRDKFNIPIYAASLEEFKEVVESDGSFIINNLQLFKGGSPLVVNHRDDAAEIGRALATTCRSVTG
VLVDAHIGDQLGHELFSRVASRAMAQAKELLDQLQFFHIVASLSLA

Supplementary Figure 2. A. Phylogenetic relationships of IAMT and CCMT from Lamiaceae. Node 5 represents the ancestral enzyme that gave rise to the CA-preferring enzymes within *Ocimum* species. B. Site-specific posterior probabilities of the ancestral sequence from Node 5. C. The primary amino acid sequence for the most-likely ancestral sequence estimated for Node 5.

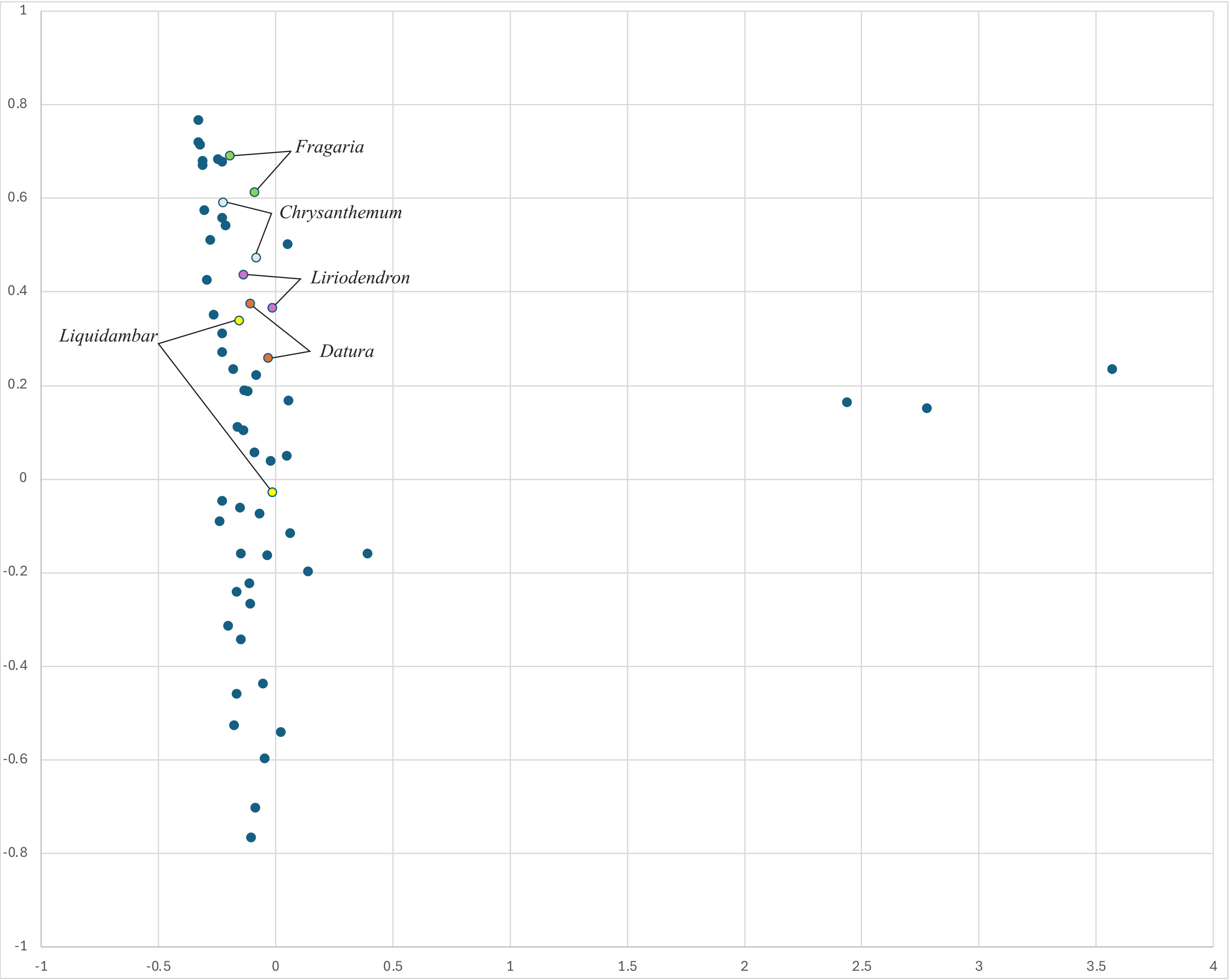

Supplementary Figure 3. Correspondence analysis shows that IAMT-type enzymes are clearly separated by Component 1 which distinguishes those that show high relative activity with CA but not other substrates. Component 2 separates the rest of the enzymes along a gradient from those that prefer IAA and show little activity with other substrates. We have assayed 5 species from each major angiosperm lineage with both the MTase-Glo luminescence assay and the 14C-SAM assay and plotted them in the correspondence analysis to evaluate to what extent results are comparable. As can be seen, the results are largely comparable with most enzymes having similar coordinates. The *Liquidambar* results are somewhat disparate but still are essentially within the same quadrant of the plot.

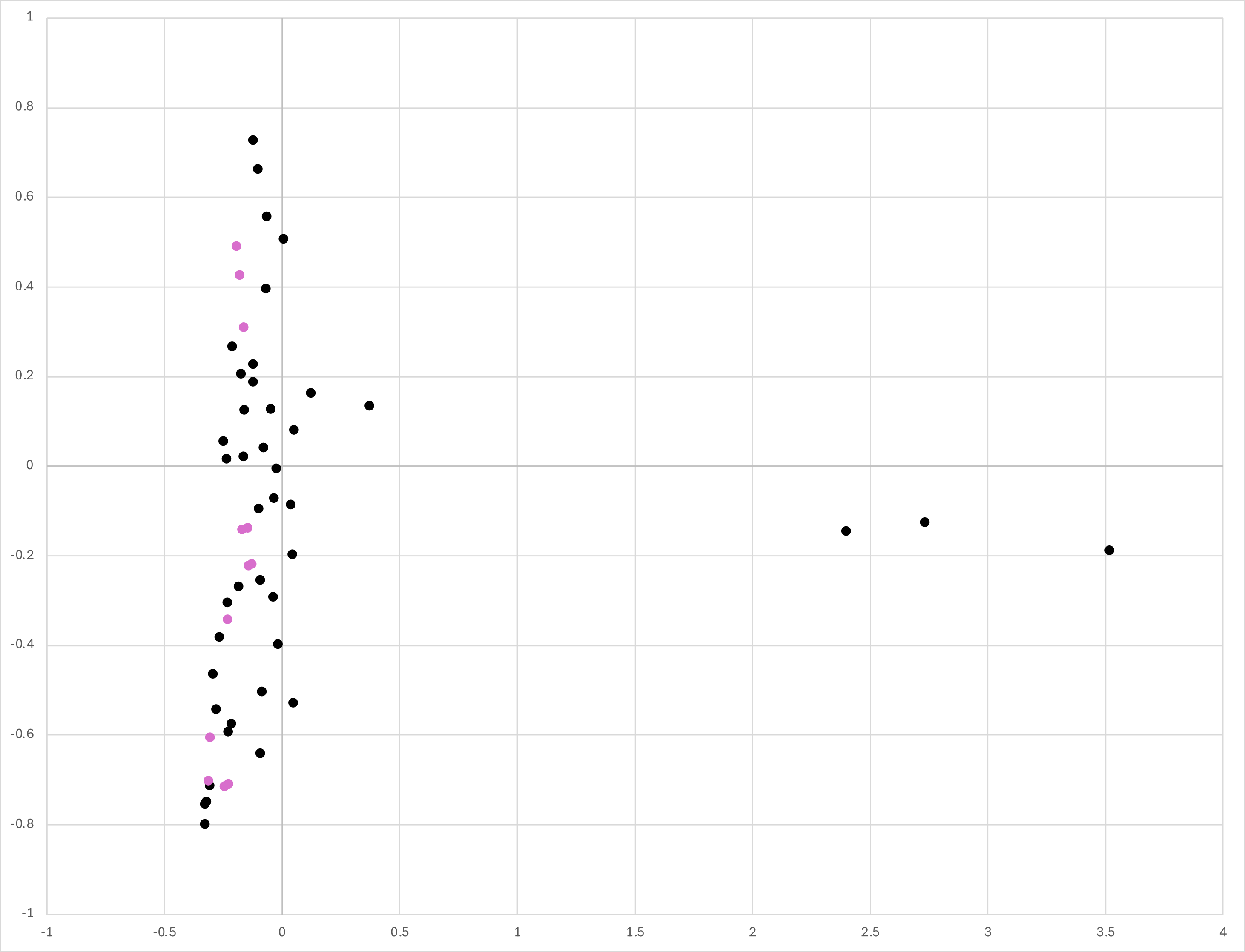

Supplementary Figure 4. Correspondence analysis shows that IAMT-type enzymes are clearly separated by Component 1 which distinguishes those that show high relative activity with CA but not other substrates. Component 2 separates the rest of the enzymes along a gradient from those that prefer IAA and show little activity with other substrates. In order to visualize the positions of data points obtained from different assays, we colored coded those obtained from MTase-GLO in black and those obtained from our radiolabeled SAM experiments in pink. As can be seen, the assays from each type do not form separate clusters as would be expected if there was an inherent bias of each method.

Supplementary Table 1. RT-PCR primers used for cDNA synthesis.

Species	Forward Primer	Reverse Primer
Antirrhinum majus	ATGAAAGGAGGCAAAGGAGAAGGA	TTAAGCTAAAGAAAGAGATGCAACAATGTGAA
Arachis hypogaea	ATGTTGCCCGCCATGGGAGACAATGTT	TTATGATTGAAGAAAAGAAAGGGATGC
Aristolochia sp.	ATGGATGCTCTGCAAGGAGACG	CTATCTTAAAGAGAGTGAGGCCAC
Beta vulgaris	ATGTCTCATAAGGGAGAAAATGTCATG	CTAGGTATTTTTTGAAGTAGTAGTTAATTTTG
Betula nigra	ATGGCTCCAAAAGGAGATAATGTTGTGG	AGCAAAAGAAAGGGATGCGACTATATGAAAG
Buddleja davidii	ATGGCTCCCATGGCAGACAATGCTGT	TTAAGCTAAAGAAAGAGATGCAACTATATG
Carica papaya	ATGGCCTCTAATGGAAATG	TTAAGCAAAAGACAAAGATGCTACTA
Catharanthus roseus	ATGGCACCAGGGGCAGGGACAG	TGCAAAAGAGAGAGATGCAACAATG
Chenopodium quinoa	ATGTCTCATAAAGGAGAGGATGTC	TTAGATATTCGTCAAAGTAGTCATTGA
Chrysanthemum x morifolium	ATGGCCCCTCCCTCCGGTGA	TCATTTAAGTGATAGTGAGGCTACAAT
Cicer arietinum	ATGGGAGATAATGTTGTAGTGTCCT	TCATTGAACTAAAGAAAGCGATGC
Citrus limon	ATGGCTCCCAAAGGAAATGATG	AAGCAAAAGAAAGGGATGCAACTATATG
Coffea arabica	ATGGCTCCTGCGGAAGAC	CTATATAGCCAGAGAAAGAGAGGC
Corylus colurna	ATGGCTCCAAAAGGAGAC	AGCAAAAGAAAGGGATGC
Cucurbita pepo	ATGGCTCCCAAGGGCGACAACG	TTATGCAAGCGAAAGCGATGCC
Datura wrightii	ATGGCTACATTGGGAGACAATAAG	CTAGCGAAGAGAAAGGGAAGCAA
Daucus carota	ATGGCTCCTTCAGCAGACAATATT	TTATGTAAAAGAAAGCGAAGCCACTAT
Foeniculum vulgare	ATGGCTCGTTCAGCAGATAATA	TTATGTGAAAGAAAGAGAAGCCACT
Fragaria virginiana	ATGGCTCCAAAGGGAGACAATGTC	TTATAAAAAAGAGAGTGAAGCCACG
Glycine max	ATGGCTCCGATGGGAAAC	TCATTGAACAAAAGAAAGGGATGC
Gossypium hirsutum	ATGGCTCCCATGGGAGACAATG	TTAAGCAAAAGAAAGCGCAACAA
Helianthus annuus	ATGGCCGCTCCCTGAGGT	TTATTTAAGAGAAAGTGAGGCTA
Humulus lupulus	ATGGCTCCCAATAAGGGTGATAATG	AGCAAAAGAAAGAGAGGCCACTATG
Ilex verticillata	ATGGCTCATATTGGAGATAACATTG	TGCAAGAGAAAGAGATGCAACTATG
Impatiens balsaminea	ATGGCTCGTTCGGTTGAC	TTATGGAAATGATAGAGATGCAACTA
Ipomoea sp.	ATGGCTACCATAGGAGACAACAAG	GTATCTACATGCATGAGAAAGAGAAAG
Jasminum sp.	ATGGCTCCAGTGGGGGAC	CTAAGCTAGAGAAAGTGCAACT
Lavandula angustifolia	ATGGCCCCGACAGCAGAAAACGTC	TTAAGCTAAAGAAAGGGATGCAACAA
Lens culinaris IAMT1a	ATGGGAGATAATGTTGTAGTGTCC	TAGAACAAAAGAAAGCGATGCAACAATGTG
Lens culinaris IAMTb	ATGGGTATGTCTCTTATGG	TTCAATAAGAGAAAGCGACGC
Liquidambar styraciflua	ATGGCTCCCAAAGGTGACAATG	CTATTCAAAAGAAAGAGATGCAAC
Malus domestica IAMT1	ATGGCTCCGAAGGGAGACAAC	TTAAGCAAGAGAAAGTGAGGCCACTATG
Malus domestica IAMT2	ATGGCTCCCAAGGGAGATAACC	TTAAGCTAGAGAAAGGGAGGCCACTATG
Mangifera indica	ATGGCTCCCAAAGGAGACGA	CTATTCAAAAGAGAGGGAGGCA
Nelumbo nucifera	ATGGCCCTCCAAGGAGATAATG	CTATGCAACAGAGGATAAGGACG
Nicotiana tabacum	ATGGCTCCCTTAGGAGACAATAA	CTACACAAGAGAAAGGGAAGCA
Ocimum basilicum IAMT	ATGGCCCCAACGCCACAA	CTAAGCTAAAGAAAGGGATGCTACAAT
Olea europaea	ATGGCTCCACTAGGGGATAATG	CTAAGCTAGAGACAGAGATGC
Paeonia sp.	ATGGTTCTTAAAGGAGATAG	CTATGCAAGAGAAAGGGATG
Papaver sp.	ATGGGTCTTCATGGGAGAGATAATG	TGAAAATGAAAGGGAAGCAACTATAT
Pelargonium x *hortorum*	ATGGCCCCCAAAGGTGACAATG	AGAAAAAGAGAGGGATGCAAC
Perilla frutescens IAMT	ATGAAGCTTGAAAGAATGCTGAGCA	TTAAGCTAAAGAAAGGGATGCAACA
Persea americana	ATGGCTACTCTCCAAGGAG	TCAATGAGAGTGAGGCTAC
Petunia hybrida	ATGGCGCCCTTAGGAGACAATAAGG	TTACTTGTATGCAAGAGAGAGGG
Sesamum indicum	ATGGCTCCTGGGGGCGACAACGTTG	AGCTAAAGAAAGGGATGCAACAATGTG
Spinacia oleracea	ATGTCTGATAAGGGAGAGAATGTTA	CTAGATATTTGTCAAAGTAGCAACTGA
Tilia cordata	ATGGCTTCCAAGGGTGAAAATG	AGCAAGAAAAAGAGATGCAAC
Viola sp.	ATGGCTCGTAAAGACAACAATG	CTAGACAAATGAGAGTGCTAC
Vitis riparia	ATGGCTCCAAGAGGAGAGAATAACATC	CTAGGCAAAAGATAGAGATGCAACTAT

Supplementary Table 2. Vectors for protein expression of each gene and method of enzyme assay implemented for each protein. GenBank Accession numbers for each cDNA used for protein expression are provided.

Species	Expression Vector	Assay type	GenBank accession #
Antirrhinum majus	ptrcHis	GC-MS	PZ206912
Arachis hypogaea	ptrcHIS	Mtase-Glo	PZ206913
Aristolochia sp.	ptrcHis	Mtase-Glo	PZ206914
Beta vulgaris	ptrcHis	Mtase-Glo	PZ206915
Betula nigra	pBAD	Mtase-Glo	PZ206916
Buddleja davidii	ptrcHIS	Mtase-Glo	PZ206917
Carica papaya	ptrcHIS	Mtase-Glo	PZ206918
Catharanthus roseus	pBAD	Mtase-Glo	PZ206919
*Chenopodium quinoa**	pBAD	GC-MS	PZ206920
Chrysanthemum x *morifolium*	ptrcHis	Mtase-Glo	PZ206921
Cicer arietinum	ptrcHis	Mtase-Glo	PZ206922
Citrus sinensis	ptrcHIS2	Mtase-Glo	PZ206923
*Coffea arabica**	pBAD	[^{14}C]-SAM	PZ206924
Cornus florida	pET28a	Mtase-Glo	PZ206925
Corylus colurna	pBAD	Mtase-Glo	PZ206926
Cucurbita pepo	ptrcHIS	Mtase-Glo	PZ206927
Datura wrightii	ptrcHis	Mtase-Glo	PZ206928
*Daucus carota**	pBAD	[^{14}C]-SAM	PZ206929
*Foeniculum vulgare**	pBAD	[^{14}C]-SAM	PZ206930
Fragaria virginiana	ptrcHis	Mtase-Glo	PZ206931
Glycine max	ptrcHIS	Mtase-Glo	PZ206932
*Gossypium hirsutum**	pBAD	[^{14}C]-SAM	PZ206933
Helianthus annuus	ptrcHIS	Mtase-Glo	PZ206934
Humulus lupulus	ptrcHIS2	Mtase-Glo	PZ206935
Ilex verticillata	pBAD	Mtase-Glo	PZ206936
*Impatiens balsaminea**	pBAD	[^{14}C]-SAM	PZ206937
Ipomoea sp.*	pBAD	[^{14}C]-SAM	PZ206938
Jasminum sp.*	pBAD	[^{14}C]-SAM	PZ206939
Lavandula angustifolia	ptrcHis	Mtase-Glo	PZ206940
Lens culinaris IAMT1a	pBAD	Mtase-Glo	PZ206941
Lens culinaris IAMTb	pBAD	Mtase-Glo	PZ206942
Liquidambar styraciflua	ptrcHis	Mtase-Glo	PZ206943
Liriodendron tulipifera	pET28a	Mtase-Glo	PZ206944
Malus domestica IAMT1	ptrcHis	Mtase-Glo	PZ206945
Malus domestica IAMT2	pBAD	Mtase-Glo	PZ206946
*Mangifera indica**	pBAD	[^{14}C]-SAM	PZ206947
Nelumbo nucifera	ptrcHIS	Mtase-Glo	PZ206948
*Nicotiana tabacum**	pBAD	[^{14}C]-SAM	PZ206949
Ocimum americanum CCMT1	pET28a	Mtase-Glo	PZ206950
Ocimum americanum CCMT2	pET28a	Mtase-Glo	PZ206951
Ocimum basilicum CCMT	pET28a	Mtase-Glo	PZ206952
Ocimum basilicum IAMT*	pBAD	[^{14}C]-SAM	PZ206953
Olea europaea IAMT*	pBAD	[^{14}C]-SAM	PZ206954
Paeonia sp.	ptrcHIS	Mtase-Glo	PZ206955
Papaver sp.	pBAD	Mtase-Glo	PZ206956
Pelargonium x *hortorum*	pBAD	Mtase-Glo	PZ206957
Perilla frutescens CCMT	pET15b	Mtase-Glo	PZ206958
Perilla frutescens IAMT	ptrcHIS	Mtase-Glo	PZ206959
Persea americana	pBAD	Mtase-Glo	PZ206960
Petunia hybrida	ptrcHIS	Mtase-Glo	PZ206961
Punica granatum	pET28a	Mtase-Glo	PZ206962
Salvia splendens CCMT	pET15b	Mtase-Glo	PZ206963
Salvia splendens IAMT	pET15b	Mtase-Glo	PZ206964
Sesamum indicum	pBAD	Mtase-Glo	PZ206965
*Spinacia oleracea**	pBAD	[^{14}C]-SAM	PZ206966
Tilia cordata	ptrcHIS2	Mtase-Glo	PZ206967
*Viola tricolor**	pBAD	GC-MS	PZ206968
Vitis riparia	ptrcHIS	Mtase-Glo	PZ206969
